# Neurotensin promotes hepatic steatosis by regulating lipid uptake and mitochondrial adaptation in hepatocytes

**DOI:** 10.1038/s41419-025-07664-3

**Published:** 2025-04-27

**Authors:** Moumita Banerjee, Jun Song, Baoxiang Yan, Haoming Wu, Shaghayegh Norouzi, Tomoko Sengoku, Savita Sharma, Teresa W. M. Fan, Eun Lee, Daheng He, Chi Wang, Jinpeng Liu, Timothy M. Schmitt, Tianyan Gao, Heidi L. Weiss, Jing Li, B. Mark Evers

**Affiliations:** 1 Markey Cancer Center, University of Kentucky, Lexington, KY USA; 2 Department of Surgery, University of Kentucky, Lexington, KY USA; 3 Redox Metabolism Shared Resource Facility, University of Kentucky, Lexington, KY USA; 4 Department of Toxicology and Cancer Biology, University of Kentucky, Lexington, KY USA; 5 Department of Pathology and Laboratory Medicine, University of Kentucky, Lexington, KY USA; 6 Bioinformatics, Biostatistics and Bioinformatics Shared Resource Facility, Markey Cancer Center, University of Kentucky, Lexington, KY USA; 7 Division of Cancer Biostatistics, Department of Internal Medicine, University of Kentucky, Lexington, KY USA; 8 Department of Surgery, University of Kansas Medical Center, Kansas City, KS USA; 9 Department of Molecular and Cellular Biochemistry, University of Kentucky, Lexington, KY USA

**Keywords:** Cell biology, Gastrointestinal diseases

## Abstract

Metabolic dysfunction-associated steatotic liver disease (MASLD) is a multifactorial disease characterized by hepatic steatosis. Mitochondrial dysfunction resulting in the incomplete digestion of surplus fat is one of the key factors that lead to hepatic steatosis but the reason for this remains unclear. We investigated the role of neurotensin (NTS), a gut hormone, in inducing maladaptive fat metabolism in steatotic liver. We identify CD36 and PGC1α, two critical drivers of MASLD, as direct NTS signaling targets in the liver. NTS upregulates CD36, a free fatty acid receptor, in hepatocytes and promotes long chain lipid uptake. Conversely, NTS inhibits PGC1α, which acts as a lipid sensor and translocates to the nucleus to activate lipid catabolism-related genes in an AMPK-dependent manner. Thus, a high fat diet decreases the fatty acid oxidation and oxidative phosphorylation capacity of the liver and hepatocytes from NTS or NTS receptor 1 (NTSR1) wild type mice; whereas NTS deficiency preserves the lipid metabolism capacity of the liver. NTS signaling is significantly upregulated in MASLD and in metabolic dysfunction-associated steatohepatitis (MASH) human liver samples when compared to normal livers, which correlates with the expression of CD36 and oxidative phosphorylation proteins. These findings provide critical mechanistic insights into the maladaptive fat metabolism noted with steatosis in mice and humans and suggest novel strategies for therapeutic intervention of MASLD, which affects nearly one-quarter of the global population.

## Introduction

Metabolic dysfunction-associated steatotic liver disease (MASLD) [1] is characterized by accumulation of more than 5% fat in hepatocytes accompanied with metabolic dysfunctions such as obesity, insulin resistance and dyslipidemia [2]. A subset of patients with MASLD can progress to metabolic dysfunction-associated steatohepatitis (MASH), manifested by liver inflammation and fibrosis [3]. Although many factors, such as diet, obesity and insulin resistance, have been proposed to play a role in MASLD pathogenesis, a lack of understanding of molecular drivers has severely impeded the development of therapeutic strategies to attenuate this disease.

According to the “multiple hit hypothesis” in MASLD, increased supply of free fatty acids (FAs) from either the diet or adipose tissue lipolysis predisposes the liver to mitochondrial dysfunction and oxidative stress [3]. The liver is a highly adaptive organ and studies show that the hepatic mitochondria can initially adjust their ability to overcome the lipid overload conditions by upregulating FA metabolism pathways [4]. However, later stages of MASLD are characterized by hepatic mitochondrial dysfunction (i.e., alteration in expression or activity of OXPHOS complexes) [5], suggesting that dysregulated mitochondrial adaptation plays a significant role in MASLD pathogenesis [6].

Neurotensin (NTS), a tridecapeptide hormone localized to the central nervous system and specialized enteroendocrine cells (N cells) in the small intestine [7], has three major receptors: NTSR1 and NTSR2 (high and low affinity G protein-coupled receptors, respectively) and the non-G protein-coupled NTSR3/sortilin-1 [8]. Increased pro-NTS (a stable 117 amino acid precursor) levels in plasma correlate strongly with obesity, MASLD development and increased risk of metabolic diseases in humans [9–11]. Notably, De Vito et al. [12] recently reported that pro-NTS levels are the strongest independent predictor of MASLD prevalence in patients. Moreover, we previously reported that global knockout of NTS in mice protects against high fat diet (HFD)-induced obesity, insulin resistance, and hepatic steatosis [13].

Lipids are the most potent stimulus for NTS secretion from the gut into the portal vein [7]. Adipose tissue-associated lymphatic endothelial cells represent another important source for NTS availability in the liver [14, 15]. This suggests that conditions implicated in MASLD development, such as increased dietary lipids and adiposity, are conducive to peripheral NTS release. However, whether NTS directly affects liver lipid metabolism and storage is unknown. Here, we investigated the mechanism by which NTS/NTSR1/NTSR3 signaling can promote hepatic steatosis under HFD conditions in mice. We show that NTS signaling promotes lipid uptake at the expense of mitochondrial bioenergetic reserve in hepatocytes. Bioenergetic reserve represents the capability of the mitochondria to adapt their metabolic parameters and mitigate the nutritional stress resulting from increased lipid supply [16]. Moreover, we demonstrate the association of this signaling pathway with steatosis in human livers and suggest that targeting NTS signaling may have broad clinical implications for the possible treatment of MASLD.

## Methods

Materials and detailed methods are provided as Supplementary Material.

## Results

### NTS signaling promotes MASLD development

**Fig. 1:**
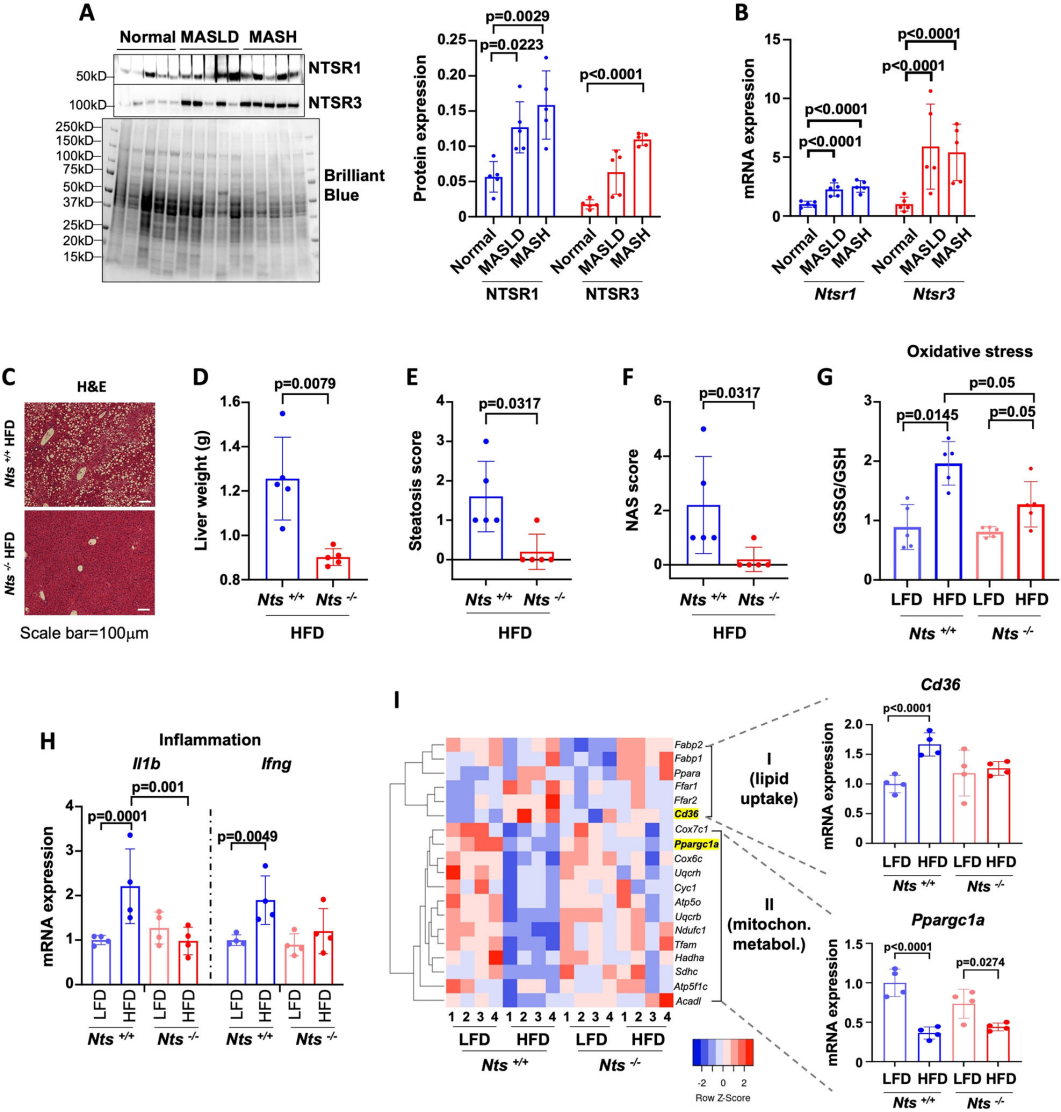
NTS signaling promotes MASLD. **A** Expression of NTSR1 and NTSR3 in normal, MASLD and MASH liver samples from human patients (western blot); *N* = 5/group. Data were normalized to total protein expression (Brilliant blue stained gel). Quantitative analysis is shown on right. **B** NTSR1 and NTSR3 gene expression (qPCR) in human normal, MASLD and MASH livers. Data were normalized to β-actin expression. **C** Female *Nts*^*+/+*^ and *Nts*^−/−^ mice were fed low-fat diet (LFD) or high-fat diet (HFD) for 28 wks. MASLD was confirmed by H&E-stained representative liver sections (10× magnification, scale bar=100 µm). **D** Liver weight, **(E)** steatosis score, and (**F**) NAS (NAFLD activity score) for *Nts*^*+/+*^ vs *Nts*^−/−^ mice fed HFD. **G** Oxidative stress in livers was compared by measuring GSSG and GSH levels (ELISA). **H** Inflammatory gene expression (*il1b* and *ifng*) in liver (qPCR, normalized to β-actin). **I** Heatmap showing expression of significantly altered metabolic genes (qPCR) in livers (log_2_fold changes, normalized to β-actin). *N* = 5 mice/group for D–G and 4 mice/group for (**H**, **I**). Data are expressed as mean ± SD, and *p* ≤ 0.05 is considered significant.

To determine the significance of NTS signaling in the pathogenesis of MASLD in humans, we compared NTSR (NTSR1, NTSR2 and NTSR3) expression in liver samples from patients with MASLD and MASH with normal livers (patient characteristics are shown in Table 1). NTSR1 protein expression was significantly higher in MASLD and MASH livers (*p* = 0.0223 and *p* = 0.0029, respectively); NTSR3 expression was higher in MASLD livers but only reached significance in MASH samples (*p* < 0.0001) (Fig. 1A). Gene expression analyses showed similar trends (Fig. 1B), whereas NTSR2 expression was not detectable. Together, these data suggest that NTS signaling is upregulated in human steatotic livers.

**Table 1:** Characteristics of patients from whom liver samples were obtained.

Sample	Sex	Age (Years)	BMI	Diabetes	Diagnosis^a^	Clinical feature
PT#1	M	61	28.02	U	Normal	
PT#2	M	38	29.24	N	Normal	
PT#3	M	53	50.58	N	Normal	
PT#4	F	18	30.83	N	Normal	
PT#5	F	55	30.66	N	Normal	
PT#6	M	52	42.37	Y	MASLD	30% steatosis, no fibrosis, no cirrhosis, no necrosis
PT#7	M	56	27.15	N	MASLD	50% steatosis (macrovesicular), no fibrosis, no cirrhosis, no necrosis
PT#8	M	58	35.98	N	MASLD	Severe steatosis
PT#9	M	20	29.55	N	MASLD	Severe steatosis
PT#10	M	44	35.67	Y	MASLD	20% steatosis (macrovesicular) + Stage 1 fibrosis
PT#11	M	20	35	N	MASH	80–90% steatosis (macrovesicular)+moderate periportal inflammation and focal active changes; necrosis present, no cirrhosis
PT#12	M	42	30.6	N	MASH	Steatohepatitis with >30% steatosis
PT#13	M	65	43.81	N	MASH	Steatohepatitis with 30% macrovesicular steatosis; mild portal inflammation
PT#14	M	36	34.03	N	MASH	Steatohepatitis with 30% macrovesicular steatosis+10% microvesicular steatosis; fibrosis (portal and bridging areas Stage 2–3)
PT#15	M	46	23.4	N	MASH	Steatohepatitis with 70% steatosis, ballooned hepatocytes, Mallory hyaline and lobular inflammation

*M* male, *F* female, *U* unknown, *Y* yes, *N* no.

^a^Normal, MASLD, MASH (*N* = 5 each).

To identify the molecular drivers of NTS-mediated maladaptive fat metabolism in the liver, *Nts*^*+/+*^ and *Nts*^−/−^ female mice were fed LFD or HFD for 28 weeks to induce MASLD. HFD significantly increased liver weight, liver weight to body weight ratio and induced steatosis, oxidative stress and inflammatory gene expression in the livers of *Nts*^*+/+*^ mice (Fig. 1C–H, Fig. S1A). Livers harvested from *Nts*^−/−^ mice were protected from the adverse effects of HFD as noted by significantly reduced lipid accumulation, oxidative stress and inflammation. Hierarchical clustering of gene expression showed that NTS signaling significantly upregulated hepatic lipid uptake and decreased mitochondrial energy metabolism under HFD conditions (Fig. 1I). Cluster of differentiation 36 (CD36), a long-chain FA transporter, is weakly expressed in normal hepatocytes but is significantly increased in MASLD livers [17]. CD36 expression was increased in livers of *Nts*^*+/+*^, but not *Nts*^−/−^, mice fed HFD. Expression of the mitochondrial biogenesis protein, PGC1α, was decreased in both genotypes under HFD conditions, although the effect was less severe in *Nts*^−/−^ livers (~67% decrease in WT vs ~39% in KO). In accordance, PGC1α transcriptional targets, *tfam*, OXPHOS and β-oxidation genes (*acadl* and *hadha*) were significantly decreased in the livers of *Nts*^*+/+*^ mice fed HFD only (Fig. 1I). Treatment of primary mouse hepatocytes with NTS over a time course revealed CD36 as an early response gene (1 h) and PGC1α as a late response gene (6 h) for NTS signaling (Fig. S1B). FA stimulation had no effect on CD36 or PGC1α expression (Fig. S1C). Together, these data suggest that NTS signaling promotes hepatic steatosis by inversely affecting two lipid metabolism pathways (i.e., lipid uptake and oxidative phosphorylation) in the liver.

Maladaptive energy metabolism in adipose tissue plays a significant role in steatosis development [18]; therefore, we examined the effects of NTS on adipose tissue lipid metabolism. Although HFD increased CD36 expression across both genotypes, it was significantly higher in adipose tissue from *Nts*^*+/+*^ mice fed HFD (Fig. S2A). This data is in accordance with their small but significantly increased body weight under the HFD condition in comparison with *Nts*^*−/−*^ mice (Fig. S2B). Consistent with the liver phenotype, PGC1α expression was decreased by HFD in white adipose tissue (Fig. S2C), and the effect was less severe in *Nts*^*−/−*^ adipose tissue (~90% decrease in WT vs 70% decrease in KO). The effect of NTS on brown adipose tissue lipid metabolism was comparatively smaller under HFD conditions (Fig. S2A,C). Taken together these data imply a systemic effect of NTS on dysregulation of lipid metabolism.

### NTS promotes lipid uptake by transcriptionally increasing CD36 expression

**Fig. 2:**
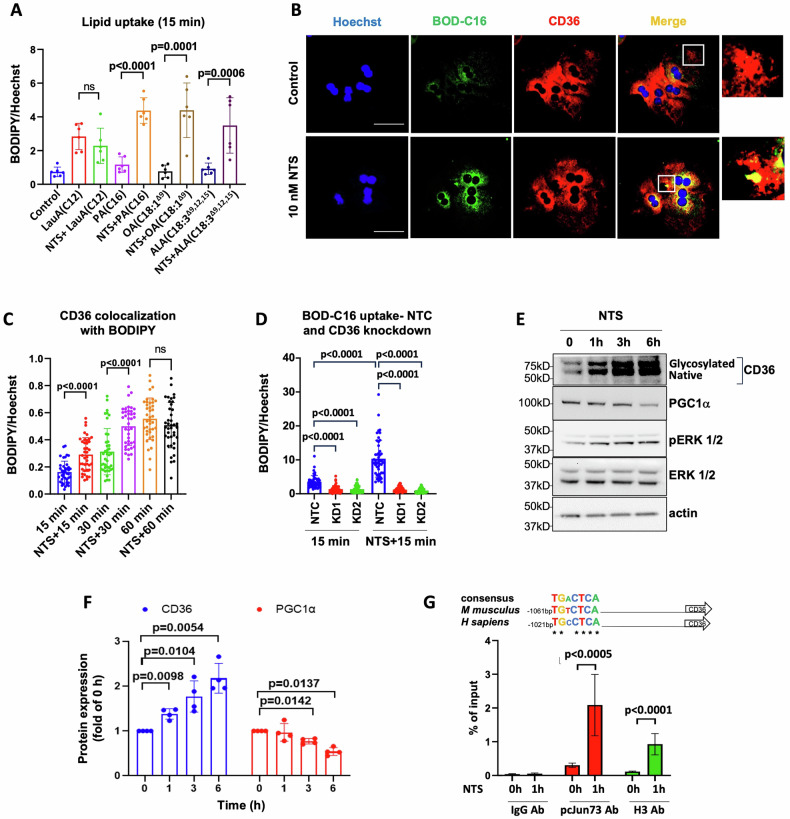
NTS promotes lipid uptake by upregulating CD36 in hepatocytes. **A** FA uptake (1 μM) at 15 min (confocal) in primary hepatocytes stimulated with or without NTS (10 nM, 1 h). Lipids were stained with BODIPY and normalized to Hoechst (nuclear stain); *N* = 6 independent experiments. **B** Representative images showing colocalization of BODIPY-labeled PA (C16) with CD36 after 15 min uptake in primary hepatocytes, with or without NTS. **C** Analyses of CD36 colocalization with BODIPY-C16 over a time course; normalized to Hoechst. *N* = 40 cells/group. **D** Lipid (BODIPY-labeled PA) uptake at 15 min was compared between control (NTC) and CD36 knockdown (KD1 and KD2) hepatocytes treated with or without NTS (10 nM, 1 h). *N* = 48 cells from two mice (2 independent experiments). NTC=non-targeted control, KD1 and KD2 = CD36 knockdown using two different CD36 targeted shRNA (lentiviral). **E**, **F** NTS induced protein expression changes (western blot); total CD36 and PGC1α expression were normalized to β-actin. *N* = 4 independent experiments. **G** Putative AP-1 binding motif in proximal promoter region of CD36 shows strong conservation with consensus sequence (top). ChIP assay (bottom) shows recruitment of pcJun 73 on the AP-1 motif in NTS-stimulated hepatocytes. Rabbit IgG and histone H3 antibodies were used as negative and positive controls, respectively. Hepatocytes isolated from C57/BL6 male mice were used for all studies. Data are expressed as mean ± SD, and *p* ≤ 0.05 is considered significant.

Increased CD36 expression is implicated in the onset of hepatic steatosis in MASLD by promoting FA uptake [19]. NTS stimulation strongly increased the uptake of several long-chain FAs such as palmitic acid (PA, C16:0, saturated), oleic acid (OA, C18:1, monounsaturated) and alpha linoleic acid (ALA, C18:3, polyunsaturated) in hepatocytes (Fig. 2A) but not the absorption of medium-chain FA, lauric acid (LauA, C12:0, saturated); indicating that NTS could promote long-chain FA uptake through CD36. Because PA is a dominant source of dietary saturated FAs [20], we selected PA for our subsequent studies.

Immunostaining (Fig. 2B, C; Fig. S3A) showed that NTS stimulation significantly promotes FA (BODIPY-labeled PA) uptake during the first 15–30 min of the lipid absorption phase. CD36 knockdown abolished NTS-stimulated FA uptake in hepatocytes (Fig. 2D, Fig. S3B), confirming CD36 to be the mediator of NTS’s effect on lipid absorption in the liver. NTS-induced CD36 upregulation was associated with activation of ERK signaling and its transcriptional effector, cJun (Fig. 2E, F; Fig. S3C, D), whereas PGC1α expression was significantly decreased. Subsequently, we identified a conserved AP-1 binding motif at 1061 bp upstream of the CD36 transcription start site in the mouse genome and show that NTS stimulation promotes pcJun73 binding at the identified AP-1 motif via ChIP assay (Fig. 2G). Notably, this sequence is conserved in the human CD36 promoter. Taken together, these data suggest a novel role for NTS signaling in increasing CD36 expression in steatotic livers.

### NTS inhibits mitochondrial energy metabolism

**Fig. 3:**
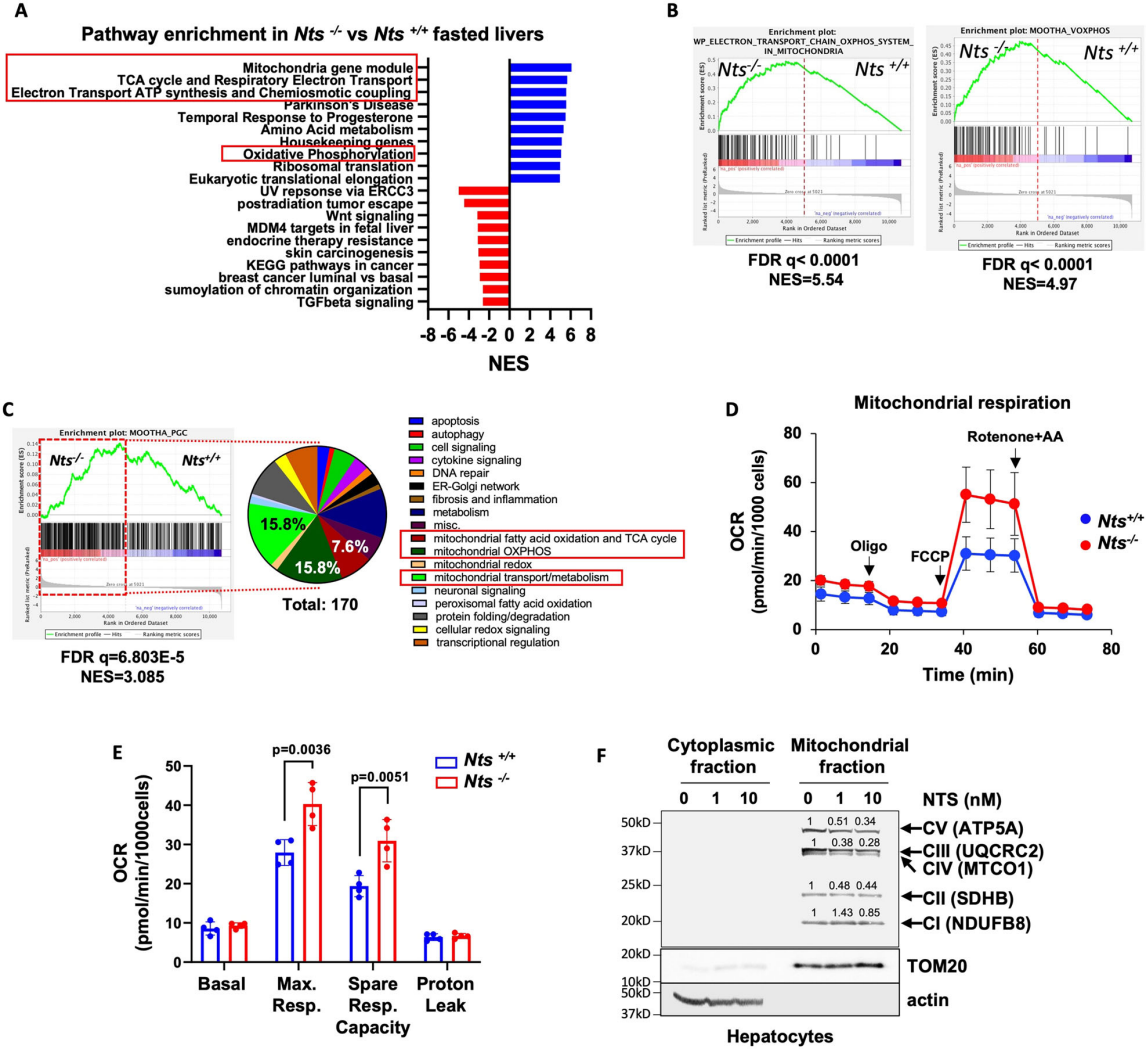
NTS compromises mitochondrial energy metabolism through OXPHOS inhibition. **A** Gene set enrichment analysis (RNAseq) showing the top 10 positively and negatively regulated pathways in fasted mouse livers (*N* = 3 mice/genotype). NES=Normalized Enrichment Score. **B** GSEA enrichment plots showing upregulation of oxidative phosphorylation in *Nts*^*−/−*^ vs *Nts*^*+/+*^ mice livers. **C** The PGC1α-regulated pathway (MOOTHA_PGC gene set) was positively enriched in liver from fasted *Nts*^−/−^ vs *Nts*^*+/+*^ mice. The upregulated genes from *Nts*^−/−^ mice within this gene set are categorized in the pie chart. The percentage of genes related to mitochondrial metabolism is labeled (red box). **D** Representative OCR of hepatocytes isolated from *Nts*^*+/+*^ and *Nts*^−/−^ mice (mito stress test). Oligomycin (oligo), FCCP and rotenone + antimycin A (AA) were added as indicated. **E** Relative mitochondrial parameters (mito stress test) of hepatocytes from 4 mice/genotype. **F** Effect of NTS treatment (16 h) on OXPHOS complex expression (western blot) in mitochondrial fractions of hepatocytes isolated from C57/BL6 male mice. TOM20 = mitochondrial fraction control, actin = cytoplasmic fraction control; expression levels were normalized to TOM20 expression and shown as fold of 0 nM NTS. Data are expressed as mean ± SD, and *p* ≤ 0.05 is considered significant.

Given that NTS inhibits PGC1α expression, we next determined whether NTS deficiency improves hepatic mitochondrial function. Because NTS has a robust effect on intestinal lipid absorption [13], we selected young adult mice fed a normal chow diet for mitochondrial activity comparisons. Livers from fasted (22 h) and fed (ad libitum) *Nts*^*+/+*^ and *Nts*^−/−^ female mice were collected for RNAseq analysis. Gene set enrichment analysis revealed the upregulation of metabolic pathways related to mitochondrial bioenergetics, OXPHOS complex and TCA cycle in livers from *Nts*^−/−^ mice under fasted, but not fed, conditions (Fig. 3A, B; Fig. S3E), which is consistent with the role of PGC1α in regulating lipid metabolism in fasted livers [21]. In accordance, PGC1α-regulated metabolic pathway (Mootha_PGC) was significantly enriched in livers from NTS-deficient mice and 40% of the upregulated genes were related to mitochondrial metabolism (Fig. 3C).

Mitochondrial function analysis (mito stress test) showed that *Nts*^−/−^ hepatocytes have significantly increased maximal respiration and spare respiratory capacity compared with *Nts*^*+/+*^ hepatocytes (Fig. 3D,E), indicating that NTS deficiency improves mitochondrial adaptive function. Spare respiratory capacity is determined by the activity of the mitochondrial respiratory chain complexes [16]. NTS treatment (16 h) decreased the expression of OXPHOS complex proteins in hepatocyte mitochondrial fractions (Fig. 3F).

Mitochondrial membrane potential, assessed by JC-1 dye aggregation ratio, is another determinant of spare respiratory capacity. NTS stimulation did not alter JC-1 ratio (Fig. S4A) in hepatocytes, suggesting that NTS signaling does not affect mitochondrial membrane potential. Taken together, these data suggest that NTS specifically decreases OXPHOS expression and activity leading to decreased mitochondrial adaptive function in the liver.

### NTS regulates mitochondrial OXPHOS function through PGC1α inhibition

**Fig. 4:**
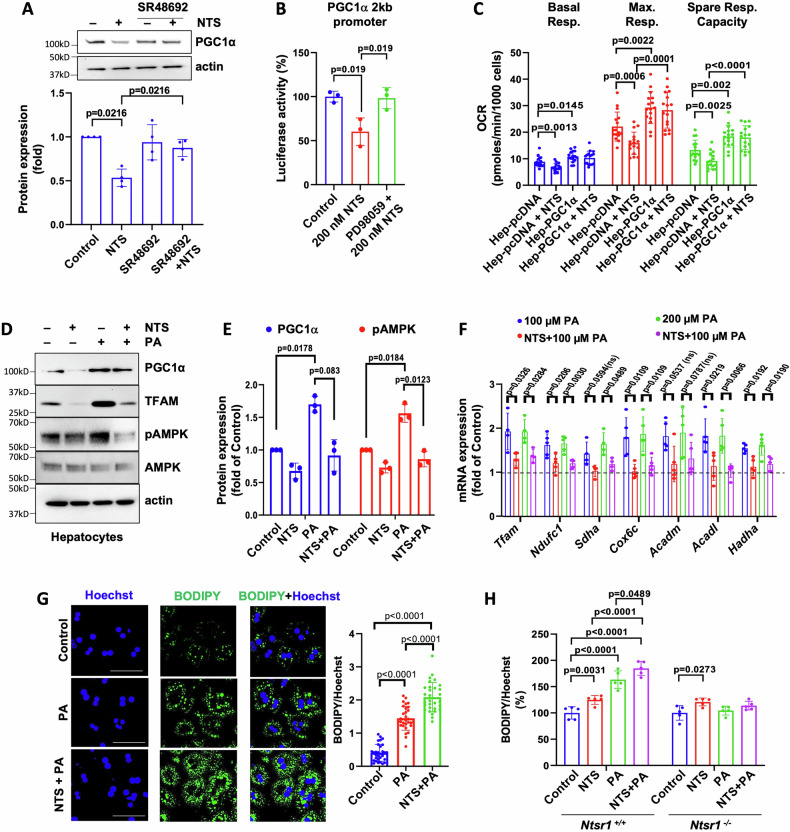
NTS regulates OXPHOS through PGC1α inhibition. **A** Effect of SR48692 (NTSR1 antagonist, 10 μM) on NTS-induced PGC1α expression in hepatocytes (C57/BL6); normalized to actin. *N* = 4 independent experiments. **B** Promoter activity of PGC1α (2 kb luciferase promoter) with NTS and PD98059 (MEK inhibitor, 1 µM) treatment in transfected HepG2 cells (Dual Glo Luciferase assay). *N* = 3. **C** Mito stress test in control (Hep-pcDNA) and PGC1α-overexpressing (Hep-PGC1α) clone 2 treated with NTS; *N* = 16 datapoints from two independent experiments. **D**, **E** Effect of NTS (10 nM) and PA (100 μM) stimulation (16 h) on protein expression (western blot) in hepatocytes (C57/BL6). PGC1α was normalized to actin and pAMPK was normalized to AMPK. *N* = 3 independent experiments. **F** Gene expression analyses in *Nts*^*+/+*^ hepatocytes treated with NTS and PA treated as in (**D**). Dashed line represents expression levels in control group; *N* = 4–5 mice; ns = not significant. **G** Lipid utilization assay. *Nts*^*+/+*^ hepatocytes were incubated with 100 µM PA for 24 h in presence/absence of NTS (10 nM), washed extensively and then incubated in lipid-free media for another 24 h. Confocal images show amount of unmetabolized lipids (BODIPY) at 48 h after lipid addition. Hoechst = nuclear stain. Scale bar = 50 μm. Quantified data from 30 cells/group are shown on the right. **H** Measurement of unmetabolized lipids by *Ntsr1*^*+/+*^ and *Ntsr1*^*−/−*^ hepatocytes at 48 h in 96-well plates (BODIPY staining was normalized to Hoechst). Representative data from two independent experiment is shown. Data are expressed as mean ± SD, and *p* ≤ 0.05 is considered significant.

To functionally connect NTS signaling with PGC1α inhibition and OXPHOS modifications, we first utilized SR48692, an NTSR1 specific antagonist [22]. SR48692 treatment reversed the NTS-induced decrease in PGC1α expression in primary hepatocytes (Fig. 4A). We further show that NTS-mediated ERK signaling regulates PGC1α at the transcriptional level since NTS treatment significantly decreased PGC1α promoter activity, which was reversed by treatment with PD98059, an MEK inhibitor (Fig. 4B)[23].

PGC1α overexpression increased OXPHOS expression (Fig. S4B,C) in HepG2 cells. NTS decreased mitochondrial function in control (Hep-pcDNA), but not in cells that overexpress PGC1α as noted by mito stress test (Fig. 4C). Conversely, PGC1α knockdown decreased the expression of several OXPHOS genes (Fig. S4D). Therefore, these studies demonstrate a role for NTS/NTSR1 signaling in the regulation of mitochondrial bioenergetics through PGC1α inhibition.

Next, we evaluated the physiological significance of PGC1α inhibition by NTS in the liver under HFD conditions. Lipid (PA) stimulation induced AMPK phosphorylation and significantly increased the expression of PGC1α and its target, TFAM, along with OXPHOS and β oxidation genes in hepatocytes (Fig. 4D–F), suggesting that hepatocytes can activate oxidative metabolism pathways through the AMPK/PGC1α axis to respond to the excess lipid influx during MASLD. This adaptive response is attenuated by NTS signaling. We then examined whether NTS-induced defects in oxidative metabolism could promote accumulation of lipid droplets in hepatocytes by inhibiting their catabolism. Hepatocytes from *Nts*^*+/+*^ mice were treated with exogenous PA for 24 h and then cultured in lipid-free media for another 24 h to allow for utilization of absorbed lipids as substrate. NTS significantly increased lipid droplet accumulation in PA-treated hepatocytes at 48 h (Fig. 4G), suggesting impaired degradation of FAs in the presence of NTS signaling. In accordance with this finding, lipid accumulation was significantly increased in hepatocytes isolated from *Ntsr1*^*+/+*^ mice (Fig. 4H), but not in *Ntsr1*^*−/−*^ hepatocytes.

### NTS inhibits PGC1α, which acts as a lipid sensor in the liver

**Fig. 5:**
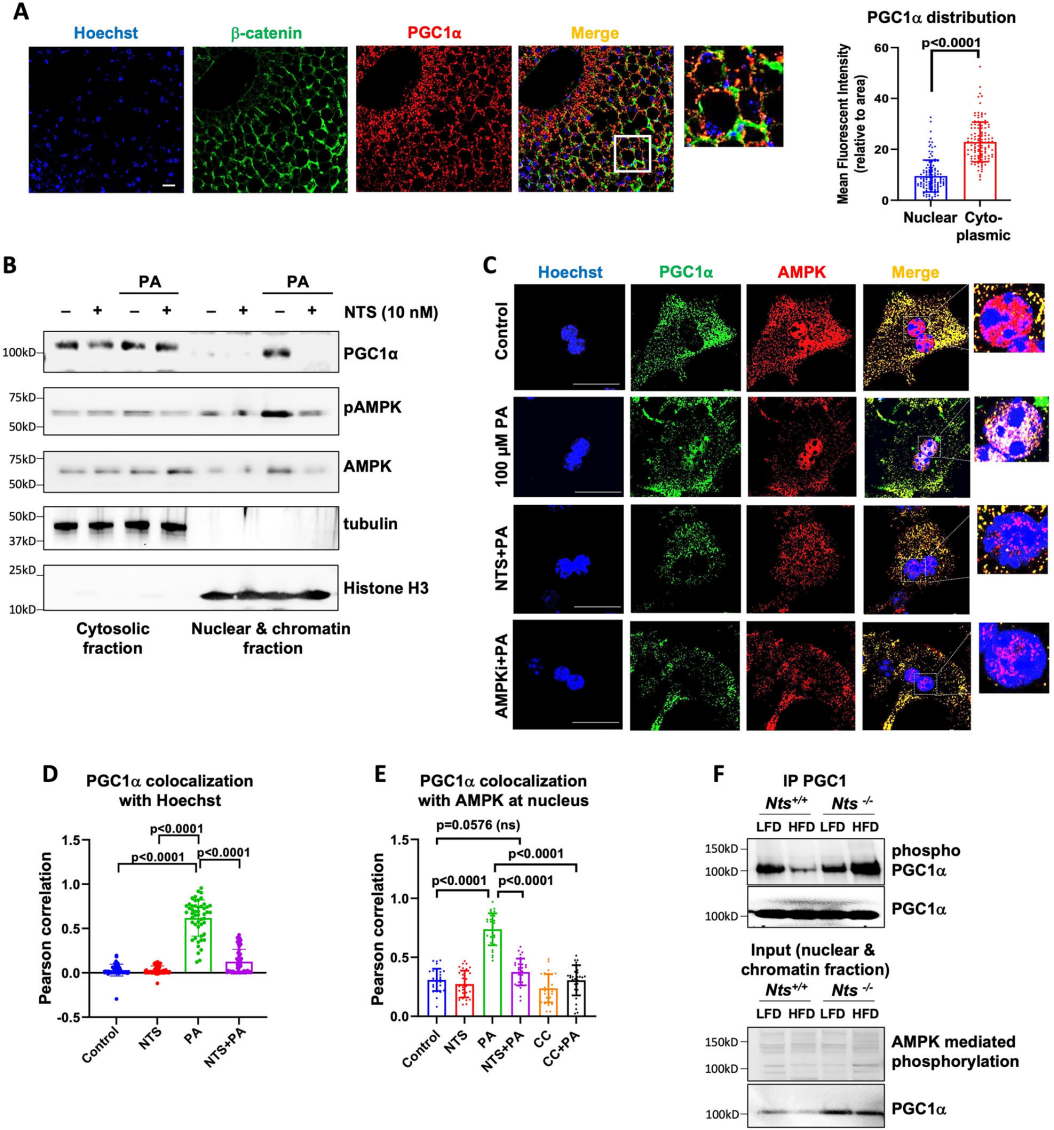
NTS attenuates the AMPK/PGC1α signaling axis. **A** Representative images of endogenous PGC1α localization in liver from *Nts*^*+/+*^ mice fed LFD (50 weeks). β-catenin = cell junction marker; Hoechst = nuclear stain. Scale bar = 50 μm. PGC1α cellular compartment distribution as normalized to nuclear or cytoplasmic area is presented on right. *N* = 120 cells. **B** Subcellular fractionation of NTS (10 nM) and PA (100 μM) treated *Nts*^*+/+*^ hepatocytes (16 h) showing PGC1α (detected using PGC1 antibody, Millipore), pAMPK and AMPK localization. Tubulin = cytosolic fraction marker; histone H3 = nuclear and chromatin fraction marker. **C** Representative images of PGC1α and AMPK localization in primary hepatocytes treated with NTS, PA and AMPKi (AMPK inhibitor = C compound, 10 μM). Nuclear colocalization is shown in inset (scale bar = 20 µm). **D** PGC1α colocalization with Hoechst (nuclear stain) was analyzed in 50 cells per group by Pearson colocalization index in hepatocytes treated as in (**C**). **E** PGC1α colocalization with AMPK at nucleus was analyzed by Pearson colocalization in 30 hepatocytes per group treated as in (**C**). **F** PGC1α was immunoprecipitated from the liver nuclear-chromatin fraction of mice fed LFD or HFD for 50 weeks and its phosphorylation was determined using an antibody that specifically identifies AMPK-mediated phosphorylation motifs. Immunoprecipitated proteins are shown on top and input on bottom. Data are expressed as mean ± SD, and *p* ≤ 0.05 is considered significant.

Collectively, our data indicate that PGC1α acts as a lipid sensor in the liver. To address this, we show that in livers and primary hepatocytes from LFD-fed *Nts*^*+/+*^ mice, PGC1α expression is predominantly localized in the cytosolic region (Fig. 5A, B). Lipid (PA) stimulation shifts PGC1α to the nuclear fraction of hepatocytes, where it can activate lipid catabolism-related genes. NTS, as well as treatment with an AMPK inhibitor (i.e., C compound) blocked PA-induced nuclear localization of PGC1α (Fig. 5B–E) supporting the notion that NTS inhibits AMPK-mediated PGC1α activation and its nuclear translocation. Moreover, immunoprecipitation analyses of PGC1α from nuclear fractions of livers from mice fed either LFD or HFD for 50 weeks (Fig. 5F) showed that HFD promotes PGC1α phosphorylation by AMPK in *Nts*^*−/−*^ liver, which was attenuated in the livers of *Nts*^*+/+*^ mice. Notably, AMPK-mediated phosphorylation is required for PGC1α activation and self-transcription using a feed forward loop in skeletal muscle [24]. Taken together, these data suggest that NTS signaling diminishes lipid catabolism capacity by inhibiting the lipid-induced AMPK/PGC1α signaling axis in liver.

### NTS signaling inhibits lipid catabolism in the liver

**Fig. 6:**
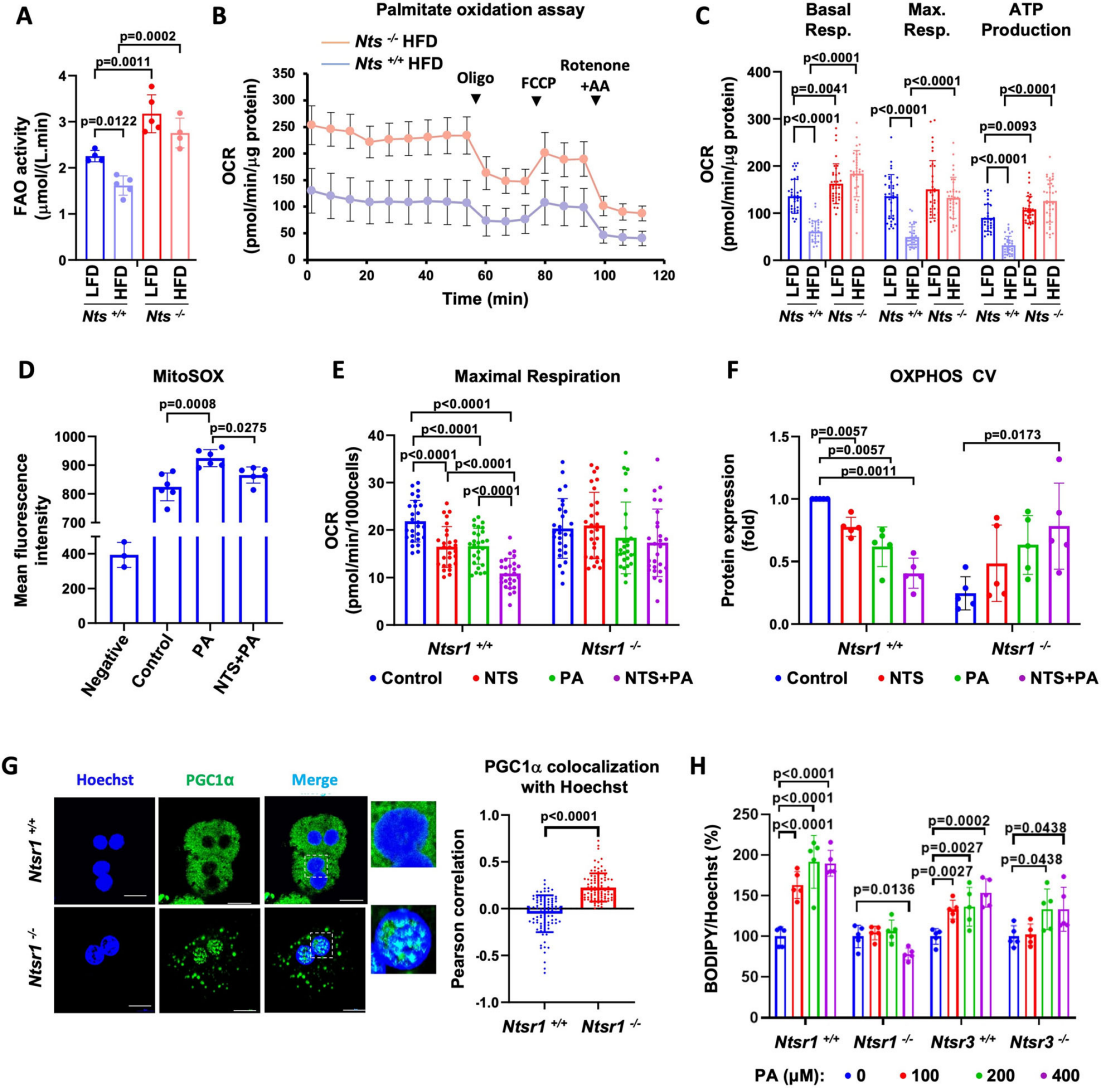
NTS/NTSR1 signaling inhibits lipid catabolism in liver. **A** Measurement of octanoate (FA) catabolism capacity in livers of *Nts*^*+/+*^ and *Nts*^−/−^ male mice fed either LFD or HFD for 23 weeks; data were normalized to protein concentration. *N* = 4–5 mice/group. **B** Representative OCR of PA oxidation by *Nts*^*+/+*^ and *Nts*^−/−^ hepatocytes isolated from mice fed HFD for 50–60 wks. Activity was normalized to protein concentration. **C** Basal, maximal respiration and ATP production from PA oxidation by hepatocytes from mice fed LFD or HFD for 50–60 weeks; *N* = 36 datapoints from 4 mice/group. **D** C57/BL6 hepatocytes were treated with 10 nM NTS (16 h) and then 100 μM PA (3 h), and MitoSOX dye uptake was measured by flow cytometry; *N* = 6 (2 repeats each from 3 independent experiments). **E** Hepatocytes isolated from *Ntsr1*^*+/+*^ and *Ntsr1*^−/−^ mice were treated with NTS and PA (50 µM) for 16 h, and mitochondrial function was measured using mito stress test. Quantitated data of maximal respiration are shown; *N* = 27 datapoints from 5 mice/genotype. **F** OXPHOS complex V expression with *Ntsr1*^*+/+*^ and *Ntsr1*^−/−^ hepatocytes treated as in (**E**) (western blot); *N* = 5 mice/genotype. Data normalized to TOM20 expression. **G** Confocal images of *Ntsr1*^*+/+*^ and *Ntsr1*^−/−^ hepatocytes stained for PGC1α and Hoechst (nuclear stain) on left. Inset shows nuclear colocalization of PGC1α; scale bar = 10 µm. Quantification of PGC1α colocalization with Hoechst on right. *N* = 90 cells from 3 mice/genotype. **H** Measurement of lipid utilization at 48 h by hepatocytes plated in 96-well plate. Unmetabolized lipids were labeled with BODIPY and normalized to Hoechst. Data are expressed as mean ± SD, and *p* ≤ 0.05 is considered significant.

β oxidation, Krebs (TCA) cycle and oxidative phosphorylation represent the three intricately related components of efficient lipid catabolism. Comparison of FAO activity in livers from *Nts*^*+/+*^ and *Nts*^−/−^ male mice fed LFD or HFD for 23 weeks (Fig. 6A) showed that HFD significantly decreased FAO capacity in livers from *Nts*^*+/+*^ mice in comparison with *Nts*^*−/−*^ livers, which was consistent with increased liver weight and downregulated PGC1α-related genes in livers of HFD-fed *Nts*^*+/+*^ mice (Fig. S5A–C). In accordance, PA oxidation assay indicated that long-term HFD feeding (50–60 weeks) strongly decreases mitochondrial OCR in the hepatocytes from male WT mice but not NTS-deficient mice (Fig. 6B,C).

Next, we measured Krebs cycle activity in hepatocytes isolated from *Nts*^*+/+*^ and *Nts*^−/−^ mice fed HFD for 23 weeks using ^13^C_6_-glucose as a tracer in stable isotope resolved metabolomics (SIRM) analysis. Results from 1D HSQC NMR analysis showed increased ^13^C incorporation into glutamate, aspartate and glutathione (GSH + GSSG) in hepatocytes of *Nts*^−/−^ mice (arrows) relative to those of *Nts*^*+/+*^ mice (Fig. S6A), which is consistent with increased Krebs cycle activity and glutathione synthesis. In addition, NTS deficiency led to increased ^13^C incorporation into the ribose moiety of adenine, guanine and uracil nucleotides and NAD^+^ (Fig. S6A, arrows), suggesting enhanced nucleotide biosynthesis via the pentose phosphate pathway, which provides the ribose precursor. These data confirm that NTS deficiency improves mitochondrial β oxidation and Krebs cycle rate and may also improve cellular redox homeostasis as the pentose phosphate pathway produces NADPH for regenerating reduced glutathione, which plays an important role in cellular antioxidant defense.

Mitochondrial ROS generated during β oxidation of FA can serve as an indicator of oxidative phosphorylation capacity [25]. NTS attenuated mitochondrial ROS generated during PA catabolism in hepatocytes as measured using MitoSOX Red dye staining (Fig. 6D).

### NTSR1 is the major NTS receptor in liver

To delineate the direct effect of NTS signaling in the liver, *Ntsr1* expression in mouse liver was confirmed using hepatocytes isolated from *Ntsr1*^*+/+*^ and *Ntsr1*^−/−^ mice (Fig. S6B). Next, we compared the effect of NTS and PA treatment on mitochondrial function of hepatocytes isolated from *Ntsr1*^*+/+*^ and *Ntsr1*^−/−^ mice. PA increased basal respiration and ATP production in hepatocytes but decreased their spare respiratory capacity (Fig. S6C). The PA-induced inhibition of OXPHOS expression was reversed by treatment with N-acetyl cysteine (NAC, an antioxidant) (Fig. S6D), suggesting that PA-induced oxidative stress inhibits the OXPHOS function of hepatocytes.

We therefore selected the lowest concentration of PA for our combination studies. NTS potentiated the inhibitory effect of PA on spare respiratory capacity in hepatocytes from *Ntsr1*^*+/+*^ mice; whereas *Ntsr1*^−/−^ hepatocytes were resistant to NTS- and/or PA-induced decrease in OXPHOS function (Fig. 6E; Fig. S7A,B). Analysis of hepatocyte mitochondrial fractions showed that NTS and PA treatment synergistically decreased the expression of OXPHOS protein complexes in hepatocytes from *Ntsr1*^*+/+*^ mice (Fig. 6F, Fig. S7C–F). Together, these data suggest that NTS signaling predisposes the liver to oxidative phosphorylation defects, which is exacerbated in the presence of lipids.

The expression of OXPHOS complex proteins was comparatively lower at basal level in hepatocytes from NTSR1-deficient mice but significantly increased with combination treatment (Fig. 6F, Fig. S7C–F). To clarify this, we show that in *Ntsr1*^−/−^ hepatocytes, PGC1α is mostly localized in the nucleus (Fig. 6G), which is likely due to their increased AMPK phosphorylation levels (Fig. S8A,B). These data are therefore consistent with our hypothesis that NTS signaling inhibits AMPK/ PGC1α signaling axis and associated mitochondrial lipid metabolism capacity of liver.

NTSR3 deletion partially rescued mitochondrial dysfunction induced by NTS treatment, and the effects were less robust than those noted with deletion of NTSR1 (Fig. S8C). Analysis of lipid utilization rate using escalating doses of PA (Fig. 6H) showed significant accumulation of unmetabolized lipid in wild type hepatocytes (*Ntsr1*^*+/+*^ and *Ntsr3*^*+/+*^). No significant lipid accumulation was observed in hepatocytes isolated from *Ntsr1*^−/−^ mice, confirming their increased metabolic rate; hepatocytes isolated from *Ntsr3*^−/−^ mice failed to metabolize the excess lipid at higher PA concentrations. These data suggest that NTS signaling through NTSR1 is the major mediator of maladaptive fat metabolism in MASLD, whereas NTSR3 most likely plays a supportive role.

### Association of NTS signaling with mitochondrial dysfunction in human fatty liver samples

**Fig. 7:**
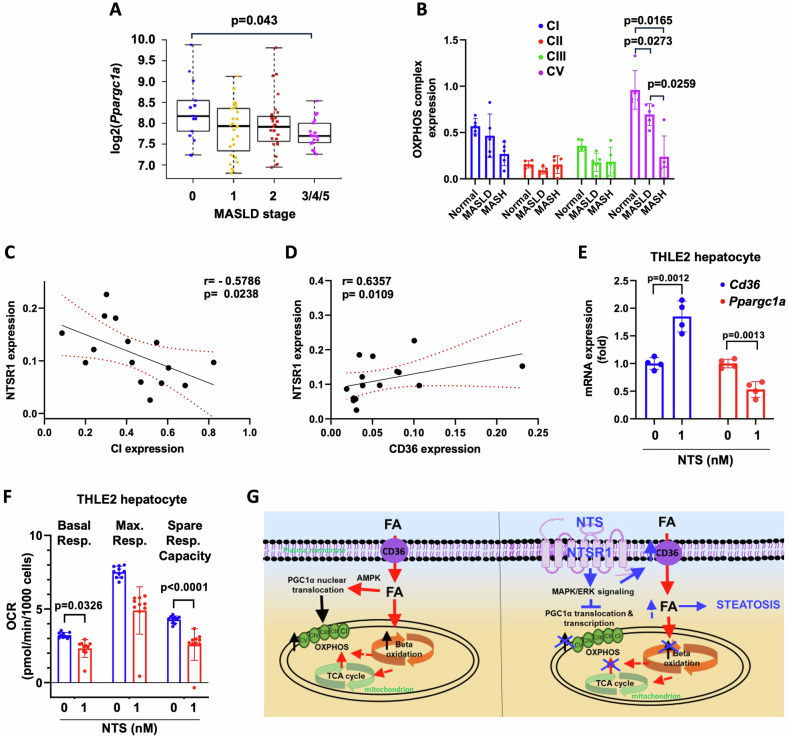
NTS signaling in human MASLD. **A** Gene expression data of PGC1α in liver biopsy samples from MASLD patients at different stages of the disease (Tukey’s HSD test) in EMBL-EBI database. **B** OXPHOS complex expression in normal, MASLD and MASH human liver samples (western blot). Data were normalized to TOM20 expression. **C** Correlation analyses of NTSR1 and OXPHOS complex I expression in liver samples from human patients. r = Spearman rho; 95% confidence interval lines are shown in red. **D** Correlation analyses of NTSR1 and CD36 expression in human liver samples. r = Spearman rho, 95% confidence interval lines are shown in red. **E** Regulation of CD36 and PGC1α expression (qPCR) by NTS in THLE2- human hepatocyte cells, normalized to β-actin *N* = 4. **F** NTS impairs mitochondrial function of THLE-2 cells (mito stress test). *N* = 10 datapoints from 2 independent experiments. **G** Schematic diagram showing liver lipid metabolism in absence (left) and presence (right) of NTS signaling and its consequence on MASLD initiation. Data are expressed as mean ± SD, and *p* ≤ 0.05 is considered significant.

We next examined whether NTS-induced mitochondrial dysfunction could be associated with MASLD in humans. Database analyses (Fig. 7A) showed that PGC1α expression is significantly decreased in the advanced stage of MASLD (Group 3/4/5 vs Group 0, *p* = 0.043; EMBL-EBI database, Accession No. E-MTAB-4856). Comparison of human liver samples (Fig. 7B; Fig. S8D) showed that expression of OXPHOS complex I, III and V was decreased in MASLD and MASH samples, although significance was only achieved for complex V (*p* = 0.0273 and *p* = 0.0165, respectively). Importantly, the expression of OXPHOS complexes I and V was significantly and inversely correlated with NTSR1 expression in human liver, whereas CD36 was positively correlated with NTSR1 expression (Fig. 7C, D; Fig. S8D–F).

To further confirm our findings, we used THLE2 cells, a model hepatocyte cell line derived from adult human liver. NTS consistently increased CD36 but decreased PGC1α expression in THLE2 cells (Fig. 7E). Moreover, NTS significantly compromised mitochondrial function in THLE2 cells (Fig. 7F), thereby establishing a multifaceted and consequential role of NTS signaling in the initiation of MASLD in humans.

## Discussion

Under obese conditions, the liver is overwhelmed with excess lipid influx, and increased mitochondrial catabolism is required for efficient disposal of the fat overload [26]. Disruption in this energy homeostasis mechanism is central for onset of MASLD. Here, we demonstrate a novel role for NTS signaling in the initiation of steatosis by promoting lipid uptake through CD36 at the expense of bioenergetic capacity of hepatocytes. We show that NTS signaling oppositely regulates two critical drivers of lipid metabolism: CD36 and PGC1α. This compromises the liver’s ability to efficiently catabolize lipids, resulting in steatosis. We also provide the first evidence that NTS signaling is significantly upregulated in livers of patients with MASLD and MASH, making this pathway an attractive target for therapeutic intervention.

CD36, a high-affinity receptor for free FAs, is implicated in the onset of steatosis in MASLD [17]. We demonstrate that NTS-induced ERK signaling rapidly upregulates hepatic CD36 expression to promote lipid uptake, as opposed to the well-established mechanism of nuclear receptor-mediated transcription which requires a longer time for activation [27]. It is plausible that NTS signaling evolved to promote rapid lipid absorption in gastrointestinal tissues to compensate for poor dietary lipid availability. In the presence of excess lipid, this mechanism can promote steatosis only if mitochondrial function becomes compromised, as NTS confers an advantage during the initial period of lipid uptake. Thus, we suggest that NTS-mediated mitochondrial dysfunction plays a predominant role in amplifying the deleterious effects of excess lipid ingestion.

PGC1α, the transcriptional coactivator, is implicated in exercise- and fasting-induced improvements in metabolic adaptation of the liver [28]. We have shown that PGC1α acts as a lipid sensor and transcriptionally activates genes involved in lipid catabolism, particularly β oxidation and oxidative phosphorylation genes. Interestingly, livers from NTS- or NTSR1-deficient mice expressed less OXPHOS proteins but showed higher fat metabolism capacity. To clarify, we showed that NTS deletion promotes PGC1α nuclear translocation through an AMPK-dependent manner, allowing for rapid transcription of oxidative metabolism genes in response to nutritional stress cues. This enhanced capability to respond to the nutritional cues [29] represents the adaptive function of the liver and is compromised by NTS signaling. Thus, when challenged with HFD conditions, NTS/NTSR1-deficient hepatocytes demonstrate better mitochondrial adaptive response. Accordingly, NTS deletion improves the FAO rate in mouse livers, whereas HFD feeding induces a progressive decline in FAO capacity in livers of *Nts*^*+/+*^ mice, with an approximate 50% reduction in hepatic FAO capacity of mice fed HFD for a longer time. Similarly, Li et al. [15] reported that NTS secreted from the lymphatic system inhibits adaptive thermogenesis in brown adipose tissue, although a direct mechanism was not identified.

In this study we identified NTS as a major regulator of PGC1α-mediated mitochondrial adaptive function in the liver and established the importance of this signaling mechanism in the pathogenesis of steatosis in mice. In human liver samples, we showed that NTS signaling and its receptors are strongly associated with increased lipid intake but compromised oxidative phosphorylation. Koliaki et al. [30] reported impaired mitochondrial biogenesis in livers of obese MASLD patients and suggested that loss of mitochondrial bioenergetic capacity facilitates MASLD progression to MASH; interestingly, oxidative damage was not noted prior to the development of MASH. Notably, PGC1α is also responsible for maintaining redox balance in tissues [31] and liver-specific PGC1α deletion exacerbates MASH phenotype in mice fed a Western diet [32]. This suggests that impaired mitochondrial biogenesis is crucial in MASLD initiation and progression to MASH in humans. NTS deletion likely improved redox homeostasis in liver, as shown by SIRM analysis, and reduced oxidative stress in livers of HFD-fed *Nts*^−/−^ mice (see Fig. 1H). Improved redox homeostasis also clarifies why *Ntsr1*^*−/−*^ hepatocytes were resistant to PA-induced oxidative damage (see Fig. 6E, F).

In MASLD, “metabolic inflexibility” of the adipose tissue is one of the primary factors leading to lipid overload conditions in the liver[18]. We found that NTS signaling likely encourages a similar state of metabolic deregulation in white adipose tissue by inversely affecting CD36 and PGC1α expression. It is noteworthy that CD36 is required for long chain FA mobilization in adipocytes [33], and increased CD36 expression was recently associated with white adipose tissue dysfunction and systemic inflammation in fasting obese individuals [34]. Interestingly, the low affinity NTS receptor, NTSR2 appears to be dominant in adipose tissue (~100-fold increase of NTSR2 mRNA expression compared with the expression of NTSR1 in white adipose tissue) (Fig. S2D,E). This suggests that the liver, which only expresses the high affinity receptor, NTSR1, would be more susceptible to metabolic fluctuations by peripheral NTS signaling. Accordingly, Li et al. [15] noted that significantly larger doses of NTS (micromolar concentrations) were required to exert its anti-thermogenic effect in brown adipose tissue.

Free FAs from adipose tissue and dietary sources are estimated to account for about 60% and 15% of liver triacylglycerols in MASLD patients, respectively, under postprandial conditions [35]. We suggest that overconsumption of dietary fat, and ensuing adiposity, stimulates excess NTS release, which promotes hepatic lipid uptake but reduces the bioenergetic capacity of hepatocytes (Fig. 7G). This results in impaired fat catabolism and steatosis. Importantly, we show a direct effect of NTS on steatosis development and suggest that the NTS/NTSR1 signaling axis represent a novel therapeutic target for MASLD. This supposition is corroborated by epidemiological data [10, 11] and findings by Wu et al. [36], who showed significant reduction in liver and body weight in HFD-fed mice when treated with a pro-NTS targeted monoclonal antibody.

### Limitations of the study

Adipose tissue–liver crosstalk plays a significant role in MASLD development. Given the well-established role of adipokines like leptin and adiponectin in MASLD development, we cannot exclude the possibility that either of these hormones was altered in the presence of NTS signaling. Specifically, the anorectic hormone leptin has been shown to modulate NTS secretion in rodent brain and to regulate feeding behavior [37]. However, despite these potential limitations, we believe that our findings provide evidence for NTS contributing a direct effect on the maladaptive lipid metabolism in the liver as noted with MASLD.

## Availability of data and materials

All relevant data are provided in the Results and Supplementary sections of the manuscript. Materials will be provided to other researchers upon reasonable request to the corresponding author (BME). RNAseq data is available through the Gene Expression Omnibus (NCBI NIH) under accession number GSE290723.

## Supplementary information

Supplemental Materials

Uncut Western Blots
