# Supplementary material for: Neurotensin promotes hepatic steatosis by regulating lipid uptake and mitochondrial adaptation in hepatocytes
Source: Cell Death Dis. 2025 Apr 27;16(1):347. doi: 10.1038/s41419-025-07664-3 (PMC12033321; doi:10.1038/s41419-025-07664-3)
Supplement: Supplementary file 1 — Supplemental Materials [file 41419_2025_7664_MOESM1_ESM.docx]

**Supplemental Materials**

[Methods 1](#_Toc195000666)

[Supplemental References 9](#_Toc195000667)

[Supplemental Figures 10](#_Toc195000668)

# Methods

***Materials.*** PGC1α (2178), phospho-p44/42 ERK/MAPK(Thr202/Tyr204) (4370), phospho-cJUN (3270), TOM20 (42406), histone H3 (4499) antibodies were from Cell Signaling Technology (Danvers, MA). OXPHOS rodent antibody cocktail (45-8099) was purchased from ThermoFisher Scientific (Waltham, MA). CD36 (NB400-144) antibody was from Novus Biologicals (Centennial, CO). PGC1 (AB3242) antibody was from Millipore (Burlington, MA). BODIPY FL (D2184), BODIPY FL-C16 (D3821) and Hoechst 33342 (H1399) dyes were from ThermoFisher Scientific. PD98059 was from Selleck Chemicals (Houston, TX). SR48692 was from Tocris (Minneapolis, MN). NTS and Liberase (5401020001) were from Sigma-Aldrich (St. Louis, MO). HepG2 and THLE-2 cells were purchased from ATCC (Manassas, VA) and cultured in Minimal Essential Media (GIBCO, 110985-080) with 10% FBS + 1% Penicillin/Streptomycin (for HepG2) and Bronchial epithelial growth media (Lonza, CC-3170) with 5 ng/ml epidermal growth factor, 70 ng/ml phosphoethanolamine, 10% FBS and 1% Penicillin/Streptomycin (for THLE-2), according to ATCC recommendations.

***Plasmids and shRNA.*** PGC1α promoter 2kb luciferase (plasmid #8887) and pcDNA 3.1 Flag-PGC1-α1 (plasmid #45501) were obtained from Addgene (Watertown, MA)^1^, and were transfected into HepG2 cells using Lipofectamine 3000 reagent (ThermoFisher Scientific). PGC1α and NTSR1 expression in HepG2 and THLE2 cells were knocked down using shRNA; RCN0000357674 for NTSR1 and TRCN0000001168 for PGC1α (Sigma-Aldrich). Protein overexpression and knockdown were verified by qPCR. PGC1α promoter activity was measured 6 h after NTS treatment using Dual Glo Luciferase Assay (Promega, E2920).CD36 expression in isolated mouse hepatocytes(C57/BL6) was transiently knocked down using two lentiviral shRNA; TRCN0000066520 and TRCN0000066522 from Sigma-Aldrich. A non-targeted control vector (shC016VN) was used as a transfection control. Briefly, hepatocytes plated on coverslips were transfected with non-targeted control or CD36 knockdown lentiviral shRNA for 16 h, allowed to recover for 24h and then utilized for lipid uptake assay. CD36 knockdown was verified by qPCR.

***Mice.*** All protocols were performed according to ethical guidelines of the Institutional Animal Care and Use Committee at the University of Kentucky. *Nts^+/+^* and *Nts*^‑/‑^ (whole body) mice ^2^ were bred from *Nts^+/-^* mice and maintained in 14 h light/10 h dark cycle with ad libitum access to food and water. *Ntsr1^+/+^* and *Ntsr1*^‑/‑^ (whole body) were bred from heterozygous B6.129P2-*Ntsr1* tmDgen/J mice (The Jackson Laboratory, Bar Harbor, MI) and housed similarly. *Ntsr3^+/+^* and *Ntsr3^‑/‑^* mice (whole body; Taconic Biosciences, Cambridge City, IN) ^3^ were bred and housed similarly. Mice were placed on low-fat diet (LFD; 10% kCal from fat, 70% kCal from carbohydrate, 20% kCal from protein containing 0.0018% cholesterol (w/w); D12450B, Research Diets, New Brunswick, NJ), HFD (60% kCal from fat, 20% kCal from carbohydrate, 20% kCal from protein containing 0.03% cholesterol (w/w); D12492, Research Diets), or normal chow diet at weaning. Age- and sex-matched littermates were used for all studies. In addition, male C57/BL6 mice (The Jackson Laboratory) fed normal chow were used for hepatocyte isolation and some mechanistic studies.

***Hepatocyte isolation.*** Primary hepatocytes were isolated by a two-step collagenase perfusion technique ^4^. Briefly, mice were anesthetized with ketamine/xylazine and livers were perfused by retrograde perfusion into the vena cava. Following Liberase digestion and Percoll (15–40%) gradient centrifugation, viable hepatocytes were plated on collagen-coated dishes at 80% confluency for 3 h in DMEM + 10% FBS + 1% penicillin/streptomycin (plating) media and then in William’s E media + 2 mM glutamine + 1% penicillin/streptomycin (maintenance media). All experiments were performed within 48 h of isolation, except for lipid utilization assays and CD36 knockdown assays which required 72 h.

***Mitochondrial fractionation.*** Cytosolic/mitochondrial fractionation was performed by the sucrose density gradient method ^5^. Briefly, hepatocytes were washed with PBS, homogenized in sucrose buffer and centrifuged at 1000 g for 10 min at 4°C to remove cell debris and nuclei. Supernatants were then centrifuged at 13000 g for 20 min to separate cytosolic (supernatant) and mitochondrial (pellet) fractions. TOM20 was used as a mitochondrial marker, whereas actin or tubulin was used as a cytoplasmic marker.

***Mito stress test.*** Mito stress test was performed using Seahorse XF96 extracellular flux analyzer. Briefly, 5000 hepatocytes were plated on collagen-coated XF96-well plates, and at indicated timepoints, oligomycin (1 µM), FCCP (1 µM) or rotenone + antimycin A (0.5 µM) was added. Oxygen consumption rate (OCR) measurements were normalized to cell count. For THLE2, 10000 cells/well were tested with similar drug concentrations. For HepG2, 15000 cells/well were plated and oligomycin (1.5 µM), FCCP (1 µM) or rotenone + antimycin A (1 µM) was used for mito stress test. Data were quantified from 2–5 independent experiments and at least 5 wells/group.

***Lipid utilization assays.*** Isolated hepatocytes in dark 96-well plates (8000 cells/well) were incubated with palmitic acid (PA) for 24 h. Cells were washed extensively and incubated in lipid-free media for another 24 h. Unmetabolized lipids were measured by BODIPY staining (neutral and nonpolar lipid stain) and counterstaining with Hoechst 33342 (nuclear stain). Fluorescence intensity of BODIPY was measured by spectrophotometer (Varioskan LUX microplate reader) and normalized to Hoechst as described previously ^5^. Hepatocytes plated on coverslips were treated similarly for immunofluorescence staining with BODIPY (neutral lipid stain) and Hoechst.

***Bioinformatics.*** Normalized whole-transcriptomic profiling array data were downloaded from the EMBL-EBI database ^6^. Disease stages 3, 4, 5 were combined into a single group to account for the relatively small samples in these groups. ANOVA with post-hoc Tukey’s HSD test was applied to the log2-transformed expressions of the gene of interest, *PPARGC1A*, to test the difference among the four disease stages (0, 1, 2 and 3/4/5). Data processing and analyses were performed using R (v. 4.0.0).

***Human samples.*** Fresh-frozen human liver samples (100 mg) were obtained from the Liver Center Biorepository at the University of Kansas Medical Center. Samples were obtained from rejected donor organs, and disease status (normal, MASLD, MASH) was determined histologically from H&E-stained sections. Liver samples were ground in liquid nitrogen for protein and RNA extraction.

***Statistical analyses.*** Descriptive statistics (mean and standard deviation) and bar graphs were generated to summarize biological and molecular markers for each genotype, diet and other experimental parameters. One-way ANOVA for multiple groups or multiple doses, or two-way ANOVA with interaction for genotype and diet experiments was utilized. Pairwise comparisons between groups were performed within the ANOVA models with adjustment for multiple testing using the Holm’s p-value adjustment. Linear mixed models were employed to model repeat measurements over time in Seahorse, Mito stress and other experiments. Two-sample *t*-tests were employed for two-group experiments. Assessment of normality assumptions and model fit were performed and appropriate data transformations or model fit adjustments were utilized. Statistical analyses were performed using SAS 9.4.

***GSH:GSSG ELISA.*** For measurement of oxidative stress, reduced (GSH) vs oxidized (GSSG) glutathione levels were assessed from snap frozen liver tissue using GSH/GSSG ratio detection kit II (Fluorometric) from Abcam (ab205811; Cambridge, United Kingdom) using the manufacturer’s recommended protocol. Data were normalized to protein concentration measured using Bradford assay.

***RNAseq analyses and gene set enrichment analyses.*** Total RNA was extracted from snap frozen liver tissues and RNAseq and gene set enrichment analyses were performed as previously described ^7^.

***Histology.*** Livers were fixed in 10% neutral-buffered formalin, embedded in paraffin, sectioned and H&E staining was performed. Evaluation of steatosis and NAS (NAFLD activity score) was performed by a pathologist blinded to treatment conditions and subsequently scored according to the NASH Clinical Research Network Scoring System as described by Kleiner et al. ^8^

***Western blot.*** Protein was extracted from snap frozen liver tissues or cells with RIPA buffer, and an equal volume of protein was separated in 4–12% NuPAGE Bis Tris gels (ThermoFisher Scientific), and western blots were performed as described by Xiong et al. ^5^.

***Real time PCR.*** Total RNA was isolated from snap frozen liver tissues or cells using the RNeasy Mini kit (Qiagen, Germantown, MD). Based upon purity, RNA from 4 mouse livers/genotype were used for cDNA synthesis and real-time PCR reactions were performed using mouse specific primers as described previously ^9^. Similar protocol was used for human primers. Primer sequences used in this study are shown in Table S1. All values were normalized to β-actin. Hierarchical clustering of gene expression change was made using Heatmapper software. RNeasy lipid tissue minikit (Qiagen #74804) was used for RNA isolation from snap frozen adipose tissues and RNA from 4 mouse adipose tissue were used for cDNA synthesis and real-time PCR as described above.

**Table S1. qPCR primers.**

## TaqMan Primers

| Gene | Species | TaqMan Probe | Company |
| --- | --- | --- | --- |
| PPARA | Mouse | Mm00440939_m1 | ThermoFisher Scientific |
| PPARGC1A | Mouse | Mm01208835_m1 | ThermoFisher Scientific |
| NRF1 | Mouse | Mm01135606_m1 | ThermoFisher Scientific |
| TFAM | Mouse | Mm00447485_m1 | ThermoFisher Scientific |
| FFAR1 | Mouse | Mm00809442_s1 | ThermoFisher Scientific |
| FFAR2 | Mouse | Mm02620654_s1 | ThermoFisher Scientific |
| FABP1 | Mouse | Mm00444340_m1 | ThermoFisher Scientific |
| FABP2 | Mouse | Mm00433188_m1 | ThermoFisher Scientific |
| IL1B | Mouse | Mm00434228_m1 | ThermoFisher Scientific |
| IFNG | Mouse | Mm01168134_m1 | ThermoFisher Scientific |
| IL6 | Mouse | Mm00446190_m1 | ThermoFisher Scientific |
| HNF4A | Mouse | Mm01247712_m1 | ThermoFisher Scientific |
| ACTB | Mouse | Mm02619580_g1 | ThermoFisher Scientific |
| PPARGC1A | Human | Hs00173304_m1 | ThermoFisher Scientific |
| CD36 | Human | Hs00169627_m1 | ThermoFisher Scientific |
| ACTB | Human | Hs01060665_g1 | ThermoFisher Scientific |

***SYBR Green Primers***

| Gene | Species | | Forward Primer (5’ – 3’) | Reverse Primer (5’ – 3’) |
| --- | --- | --- | --- | --- |
| ACADM | Mouse | | AACACTTACTATGCCTCGATTGCA | CCATAGCCTCCGAAAATCTGAA |
| ACADL | Mouse | | TTTCCTCGGAGCATGACATTTT | GCCAGCTTTTTCCCAGACCT |
| HADHA | Mouse | | TGCATTTGCCGCAGCTTTAC | GTTGGCCCAGATTTCGTTCA |
| COX6C | Mouse | | GCGTCTGCGGGTTCATATTG | TCTGCATACGCCTTCTTTCTTG |
| COX7A2 | Mouse | | GCTGGCCCTTCGTCAGATT | GGCATCCCATTATCCTCCTGAA |
| COX7C | Mouse | | ATGTTGGGCCAGAGTATCCG | ACCCAGATCCAAAGTACACGG |
| CYC1 | Mouse | | CAGCTTCCATTGCGGACAC | GGCACTCACGGCAGAATGAA |
| NDUFA1 | Mouse | | ATGTGGTTCGAGATTCTCCCC | CCTGTGGATGTACGCAGTAGC |
| NDUFA9 | Mouse | | GATTGTGGCCACTGTGTTTGG | CTCCAGCTTCCTTGGACAGT |
| NDUFS6 | Mouse | | TTCGGTTTGTAGGTCGTCAGA | CCATCGCACGCTATCACCC |
| NDUFC1 | Mouse | | GTAGTGCTGCGCTCGTTTTC | CCAACCAGTTAGGTTTGGCAT |
| NDUFA3 | Mouse | | ATGGCCGGGAGAATCTCTG | AGGGGCTAATCATGGGCATAAT |
| SDHA | Mouse | | GCGGTGGTCACCTTGATCC | CCTCTGTAGAAGCGTCTGAATG |
| SDHB | Mouse | | ACAGCTCCCCGTATCAAGAAA | GCATGATCTTCGGAAGGTCAA |
| SDHC | Mouse | | CTGTTGCTGAGACACGTTGGT | ACAGAGGACGGTTTGAACCTA |
| SURF1 | Mouse | | AAGCGGAAGATGACTCCTTTCT | TTCACTGGCCTATACTCCAGATT |
| URCRB | Mouse | | GGCCGATCTGCTGTTTCAG | CATCTCGCATTAACCCCAGTT |
| UQCRH | Mouse | | GTGGACCCCCTAACAACAGTG | CGGGAAGACACGCGATTATCA |
| UQCRC1 | Mouse | | GTTAGCCTGCTGGACAACG | CTTGATGTAGTAAGCTGTGTGC |
| NTSR1  NTSR2 | Mouse  Mouse | | TGGCTACTATTTCCTGCGAGA  TTCACCGCGCTCTATTCGC | TGCGGGACATGAGGGTCTT  AGGGGTAGTGGGACCACAC |
| PPARGC1B | Mouse | | CTTGGCTGCGCTTACGAAGA | GAAAGCTCGTCCACGTCAGAC |
| ATP5O | Mouse | | TCTCGACAGGTTCGGAGCTT | AGAGTACAGGGCGGTTGCATA |
| ATP5F1 | Mouse | | AGTTCCTTTACCCTAAGACTGGT | TTCATGCTCGACTGCTTTACTT |
| ACTB  NTSR1  NTSR2  NTSR3 | Mouse  Human  Human  Human | | GGACGACATGGAGAAAATCTGGCA  GTGGTCATCGCCTTTGTGGT  GCGCTCTACGCACTCATCTG  GAAGTCGTGGAGGAAGAATCTTT | GTAGATGGGCACAGTGTGGGTG  GAACGGAGTCCACTGCTCAT  GTGGAACCACACGAAGCTGTA  TGGTGTTGTCTGATCCCCATT |
| ***ChIP Mouse Primers (SYBR Green)*** | | | | |
| FWD (5’ – 3’) | | TGCTATCAGCTGTGTATGGGT | | |
| REV (5’ – 3’) | | GCACTGCTCTGACTAGGCAA | | |

***Immunostaining.*** For PGC1α localization, hepatocytes were plated on collagen-coated coverslips and fixed with 4% paraformaldehyde/PBS after either NTS or PA treatment. Following blocking and permeabilization, cells were immunostained with PGC1α antibody (Cell Signaling Technology, 2178), AMPKɑ2 antibody (Santa Cruz, sc-19391), anti-rabbit Alexa Fluor 488 secondary antibody (ThermoFisher Scientific, 11008) and anti-goat Alexa Fluor 594 antibody (ThermoFisher Scientific, ab150132). Hoechst 33342 was used to stain nuclei. CD36 immunostaining (ab23680) was performed in a similar manner after fixation with ice cold methanol. Confocal images were obtained using an Olympus FV1000 microscope fitted with a camera. Data were analyzed using ImageJ Fiji software (NIH) and raw values were normalized to Hoechst staining. Formalin-fixed liver sections were blocked (10% donkey serum + 1% BSA), immunostained with anti-PGC1 (Millipore, ab3242) and β-catenin (Santa Cruz, sc-1496), respectively, after deparaffinization and antigen retrieval (citrate buffer) steps. Sections were then counterstained with anti-rabbit Alexa Fluor 594, anti-goat Alexa Fluor 488 antibodies and Hoechst (nuclear stain). Slides were washed with 10 mM CuSO_4_ and 50 mM NH_4_Cl solution to reduce autofluorescence and confocal images were taken after mounting with Prolong Gold Antifade reagent. PGC1 nuclear or cytoplasmic distribution in liver sections was analyzed by Cyt/Nuc macro in ImageJ and fluorescence intensity was normalized to respective nuclear or cytoplasmic areas from 4 images.

***JC-1 staining.*** Mitochondrial membrane potential was measured by a JC-1 membrane potential assay kit (Abcam, ab113850) in a 96-well plate format using the manufacturer’s recommended protocol.

***Cytosolic and nuclear-chromatin fractionation and immunoprecipitation.*** NE-PER nuclear and cytoplasmic extraction kit (ThermoFisher, #78833) were used for fractionation of hepatocytes and fresh liver. Chromatin pellet was homogenized with a probe sonicator (4 min) to extract chromatin-bound proteins. Tubulin was used as cytosolic marker and histone H3 expression was used as nuclear and chromatin fraction marker. Livers were collected from mice after cervical dislocation, homogenized and nuclear fractions were isolated by NE-PER kit. Nuclear fractions were further sonicated with a probe sonicator (4 min) and resulting nuclear and chromatin proteins were immunoprecipitated with PGC1 antibody (1:100 dilution, Millipore, ab3242) overnight. Next day, antibody was pulled down with protein A/G agarose resin for 4 h and then immunoprecipitated proteins were resolved by western blot for detection of AMPK-specific phosphorylation using antibody (Cell Signaling, #5759). Blots were further probed for total PGC1α (Cell Signaling, #2178).

***CHIP assay.*** Hepatocytes were plated in two 10 cc dishes, treated and then crosslinked with formaldehyde for 10 min, quenched with glycine and lysed in CHIP lysis buffer according to X-CHIP protocol (Abcam). Proteins were precipitated (16 h) from sonicated chromatin using pcJUN73 antibody (Cell Signaling, #3270), rabbit IgG control antibody (Cell Signaling, #2729) or histone H3 antibody (Cell Signaling, #4620) at 1:50 ratio. Antibodies were precipitated with protein A/G agarose resin, washed extensively, reverse crosslinked with RNase A and proteinase K enzymes in elution buffer (1% SDS, 100 mM NaHCO_3_). After purification, bound DNA fragments were analyzed by qPCR using primers spanning the entire promoter region (+100 to –1500 bp). Primers were designed from University of California Santa Cruz genome browser (genome.ucsc.edu) using the mouse genomic DNA sequence (GRCm39/mm39) as reference.

***Flow cytometry.*** Hepatocytes were treated overnight with or without NTS (10 nM), and then 100 µM PA for 3 h. Cells were washed extensively and labeled with MitoSOX Red (1 µM) in warm media for 30 min in cell culture incubator. Cells were then washed extensively and MitoSOX uptake was measured within 1 h (BD FACSymphony A3 Cell Analyzer) using the BB630 channel.

***FAO assay.*** Octanoate β oxidation assay was performed in liver tissues using the fatty acid oxidation assay kit (Biomedical Research Service, University of Buffalo, Buffalo, NY, E-141) according to the manufacturer’s recommendation. Palmitate oxidation assay (Seahorse XF96) was performed using 200 µM PA, 4 µM etomoxir or BSA (control) in the presence of oligomycin (1 µM), FCCP (1 µM) or rotenone + antimycin A (0.5 µM) according to the manufacturer’s recommended protocol. Data were normalized to protein concentration.

***BODIPY-C16 uptake.*** Hepatocytes were plated on collagen-coated coverslips and pretreated with NTS for 1 h. Next, BODIPY-C16 (1 µM) was added directly to the media; at indicated timepoints, coverslips were washed and fixed with 4% paraformaldehyde in PBS. Coverslips were stained with Hoechst and mounted using Prolong Gold anti-fade reagent (ThermoFisher Scientific). Images were obtained using FV1000 Olympus confocal microscope using 20 × or 40 × (oil) objective. Data were quantified by ImageJ Fiji software (NIH) and normalized to Hoechst staining.

***Metabolomics.*** Analysis of Krebs cycle activity in hepatocytes via 1D HSQC NMR was performed using ^13^C_6_-Glucose as tracer as previously described ^10^.

# Supplemental References

1. Handschin C, Rhee J, Lin J, Tarr PT, Spiegelman BM. An autoregulatory loop controls peroxisome proliferator-activated receptor gamma coactivator 1alpha expression in muscle. *Proc Natl Acad Sci U S A* 2003, **100**(12)**:** 7111-7116.

2. Dobner PR, Fadel J, Deitemeyer N, Carraway RE, Deutch AY. Neurotensin-deficient mice show altered responses to antipsychotic drugs. *Proc Natl Acad Sci U S A* 2001, **98**(14)**:** 8048-8053.

3. Li J, Wang Y, Matye DJ, Chavan H, Krishnamurthy P, Li F*, et al.* Sortilin 1 Modulates Hepatic Cholesterol Lipotoxicity in Mice via Functional Interaction with Liver Carboxylesterase 1. *J Biol Chem* 2017, **292**(1)**:** 146-160.

4. Charni-Natan M, Goldstein I. Protocol for Primary Mouse Hepatocyte Isolation. *STAR Protoc* 2020, **1**(2)**:** 100086.

5. Xiong X, Hasani S, Young LEA, Rivas DR, Skaggs AT, Martinez R*, et al.* Activation of Drp1 promotes fatty acids-induced metabolic reprograming to potentiate Wnt signaling in colon cancer. *Cell Death Differ* 2022, **29**(10)**:** 1913-1927.

6. Hoyles L, Fernandez-Real JM, Federici M, Serino M, Abbott J, Charpentier J*, et al.* Molecular phenomics and metagenomics of hepatic steatosis in non-diabetic obese women. *Nat Med* 2018, **24**(7)**:** 1070-1080.

7. Rock SA, Jiang K, Wu Y, Liu Y, Li J, Weiss HL*, et al.* Neurotensin Regulates Proliferation and Stem Cell Function in the Small Intestine in a Nutrient-Dependent Manner. *Cell Mol Gastroenterol Hepatol* 2022, **13**(2)**:** 501-516.

8. Kleiner DE, Brunt EM, Van Natta M, Behling C, Contos MJ, Cummings OW*, et al.* Design and validation of a histological scoring system for nonalcoholic fatty liver disease. *Hepatology* 2005, **41**(6)**:** 1313-1321.

9. Li J, Song J, Zaytseva YY, Liu Y, Rychahou P, Jiang K*, et al.* An obligatory role for neurotensin in high-fat-diet-induced obesity. *Nature* 2016, **533**(7603)**:** 411-415.

10. Reyes-Caballero H, Rao X, Sun Q, Warmoes MO, Lin P, Sussan TE*, et al.* Air pollution-derived particulate matter dysregulates hepatic Krebs cycle, glucose and lipid metabolism in mice. *Sci Rep* 2019, **9**(1)**:** 17423.

# Supplemental Figures

**
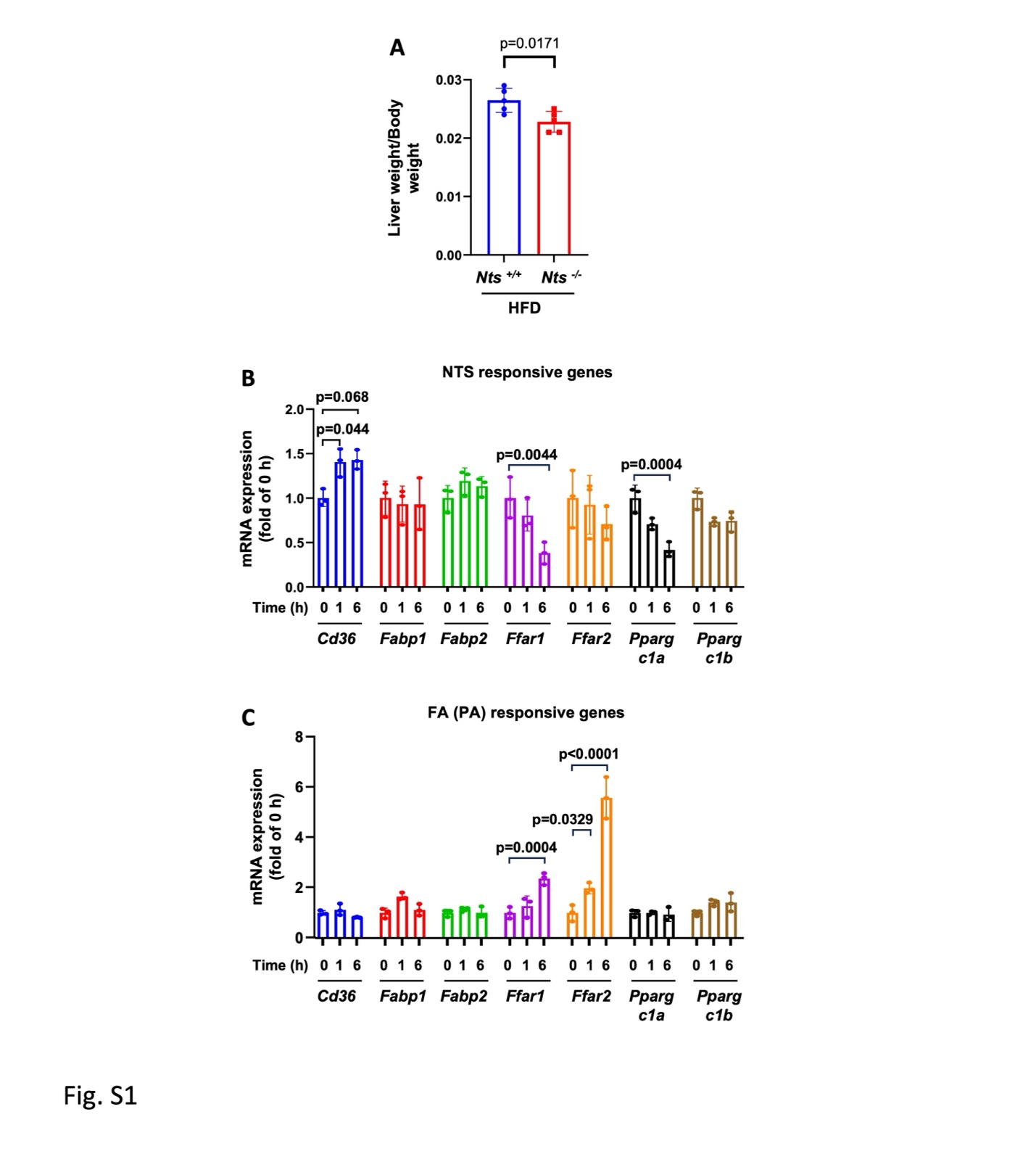
**

**Figure S1. NTS promotes MASLD by regulating CD36 and PGC1α transcription. (A)** Liver weight to body weight ratio of livers from *Nts^+/+^* and *Nts*^‑/‑^ female mice fed HFD for 28 wks. **(B)** Time course of NTS-induced gene expression (qPCR) in isolated mouse hepatocytes; mRNA expression was normalized to β-actin. N = 3 independent experiments. **(C)** Time course of PA-induced gene expression (qPCR) in hepatocytes; mRNA expression was normalized to β-actin. N = 3 independent experiments. Data are expressed as mean ± SD, and p ≤ 0.05 is considered as significant.


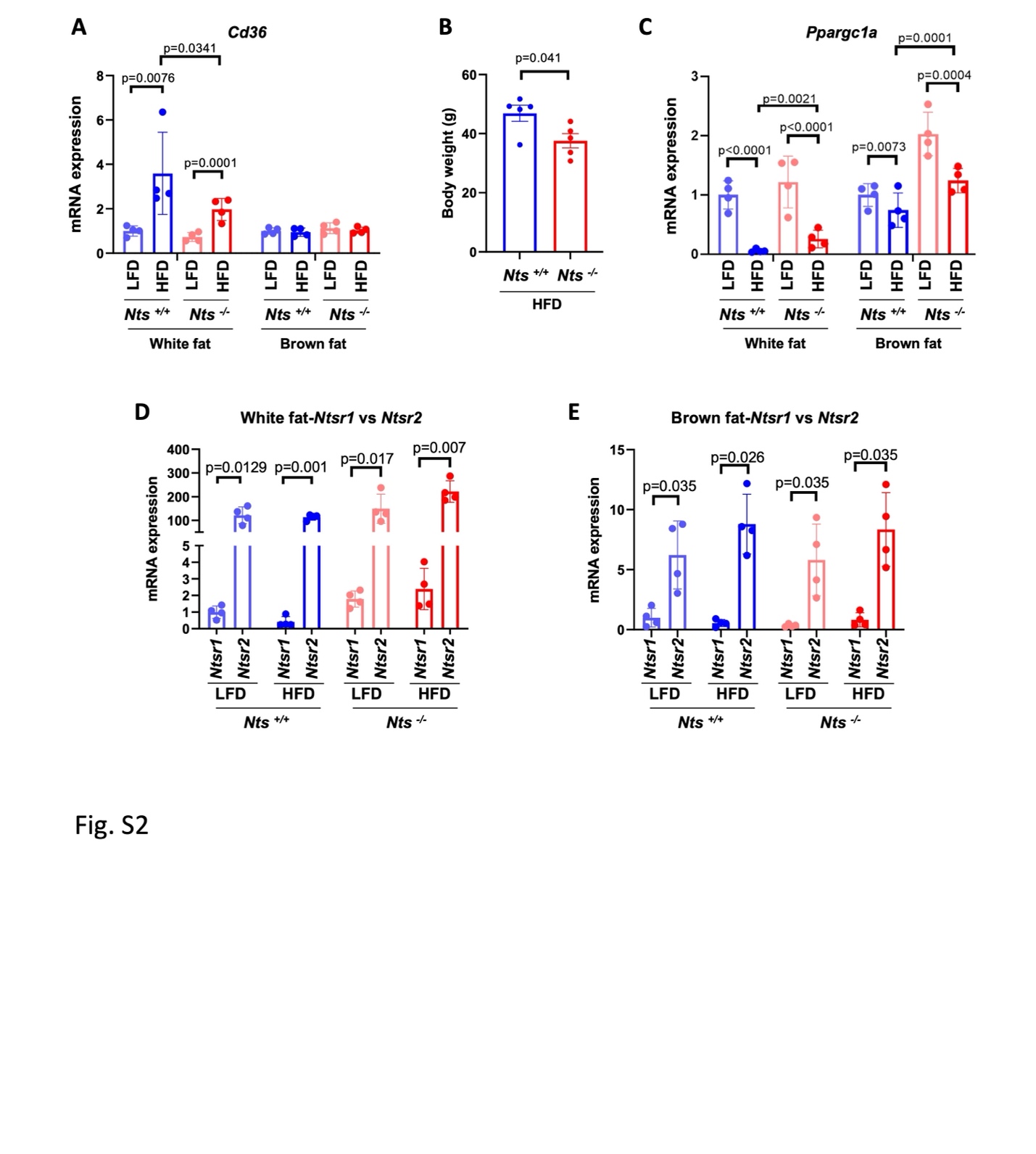


**Figure S2. Effect of NTS signaling on adipose tissue.** **(A)** CD36 gene expression (qPCR) in white adipose tissue (epididymal) and brown adipose tissue from 28 wks LFD or HFD fed *Nts^+/+^* and *Nts^-/-^* mice (female). Data was normalized to β-actin. N=4 mice/group. **(B)** Comparison of body weight in mice fed HFD for 28 wks as in (A). N=5 mice/group. **(C)** PGC1α gene expression(qPCR) in same group of mice as in (A). Data was normalized to β-actin **(D)&(E)** Comparison of NTSR1 vs NTSR2 expression in white and brown fat in same group of mice as in (A), normalized to β-actin. N=4 mice/group. Data are expressed as mean ± SD, and p ≤ 0.05 is considered as significant.


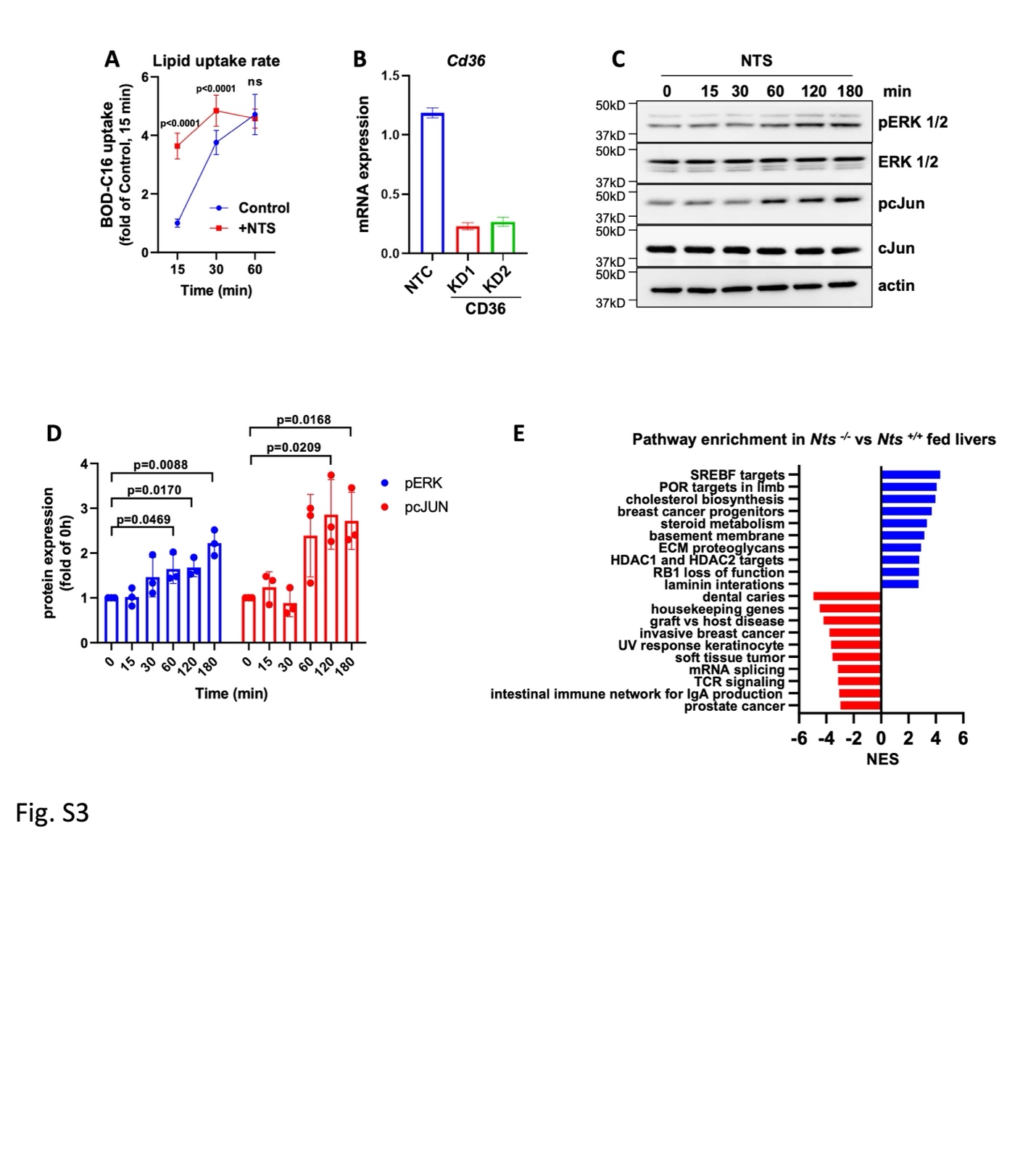


**Figure S3.** **Effect of NTS signaling on lipid uptake and metabolism. (A)** Time-dependent BODIPY-C16 uptake in hepatocytes isolated from WT mice in the absence or presence of NTS (10 nM, 1 h). Data were analyzed by ImageJ and normalized to Hoechst (nuclear stain); N = 6 fields (each containing 20–30 cells). **(B)** CD36 expression was transiently knocked down in hepatocytes isolated from 2 mice (C57/BL6) using lentiviral shRNA**.** Efficiency of knockdown using two different shRNA (KD1 and KD2) was verified by qPCR and normalized against β-actin. NTC= non-targeted control transfected hepatocytes, KD1 and KD2= CD36 knockdown hepatocytes. **(C)** Representative western blots showing the effect of NTS stimulation on ERK signaling pathways. **(D)** Densitometric quantification of pERK and pcJUN expression over a time course following NTS treatment; N = 3 independent experiments. **(E)** Pathway enrichment analysis (RNAseq) of livers from female *Nts*^‑/‑^ or *Nts^+/+^* mice under normal chow fed conditions; NES=Normalized Enrichment Score. N = 3 mice/group. Data are expressed as mean ± SD, and p ≤ 0.05 is considered as significant.


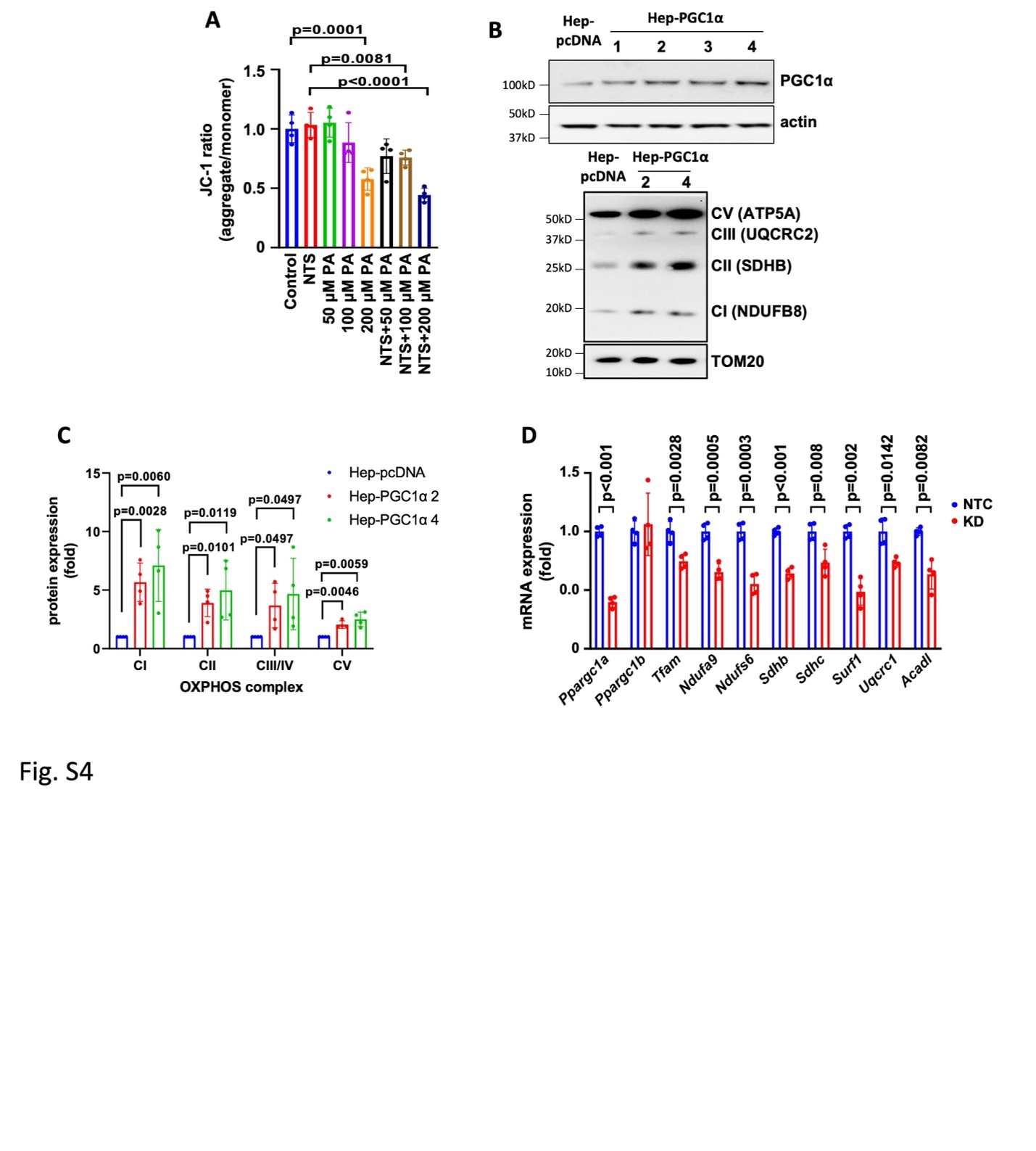


**Figure S4. NTS inhibits oxidative metabolism genes. (A)** Quantitative analysis of mitochondrial membrane permeability in hepatocytes treated overnight with NTS and/or PA as measured by JC-1 staining; N = 5 independent experiments. **(B)** Effect of PGC1α overexpression (top) on expression of OXPHOS complexes (bottom) in the mitochondrial fraction of HepG2 (western blot). **(C)** OXPHOS expression in (B) were normalized to TOM20. N=4 independent experiments. **(D)** Effect of PGC1α knockdown (siRNA) on gene expression (qPCR) in HepG2 cells; mRNA expression normalized to β-actin. NTC=non-targeted control, KD=PGC1α knockdown; N = 4 independent experiments. Data are expressed as mean ± SD, and p ≤ 0.05 is considered as significant.


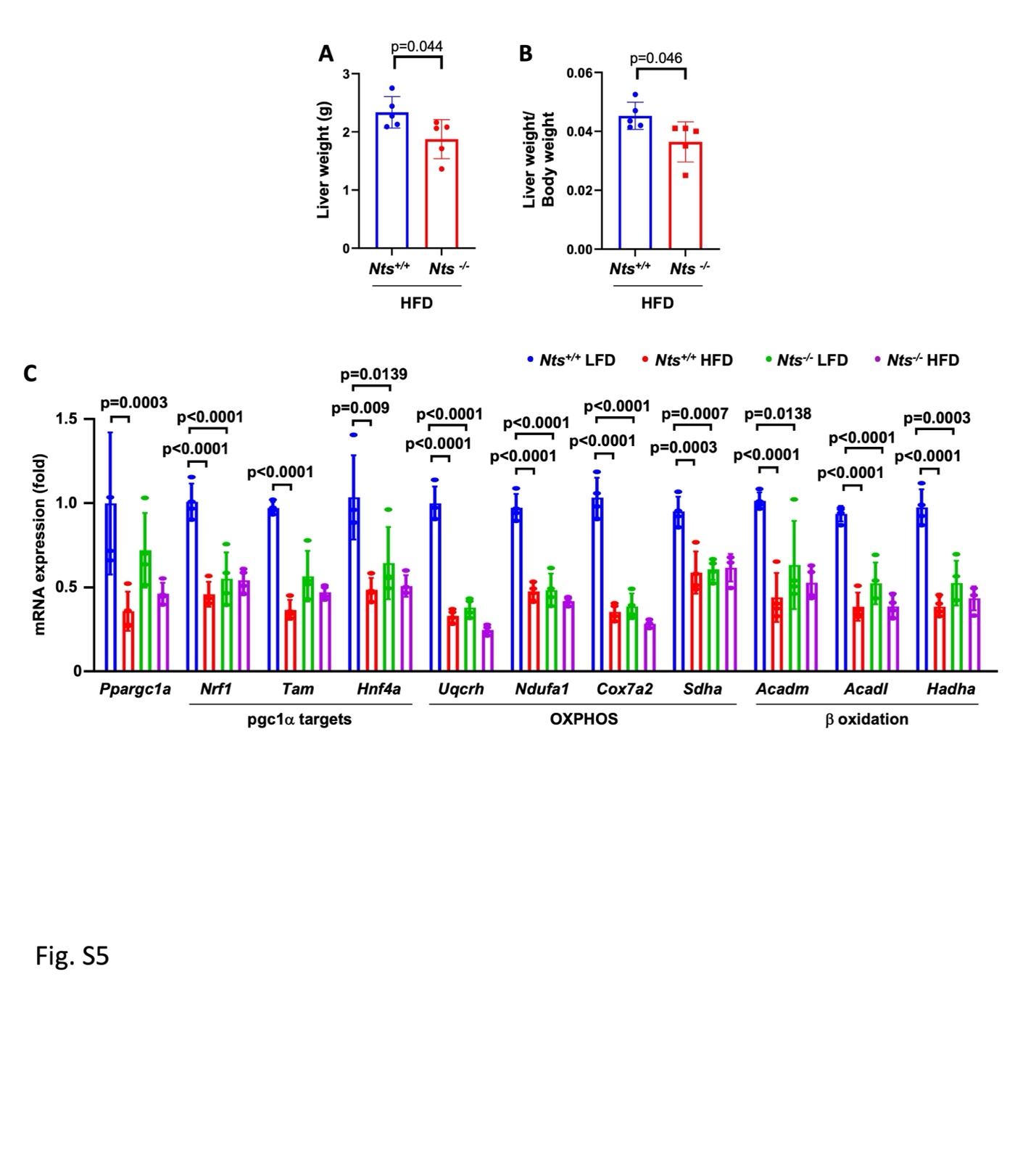


**Figure S5. Effect of NTS knockout in livers of male mice fed LFD or HFD for 23 wks. (A,B)** Comparison of liver weight and liver weight to body weight ratio in *Nts^+/+^* and *Nts^-/-^* male mice fed HFD for 23 wks. N=5 mice/group. **(C)** Gene expression analyses of livers from *Nts^+/+^* and *Nts*^‑/‑^ male mice fed LFD or HFD for 23 weeks, normalized to β-actin; N = 4 mice/group. Data are expressed as mean ± SD, and p ≤ 0.05 is considered as significant.

**
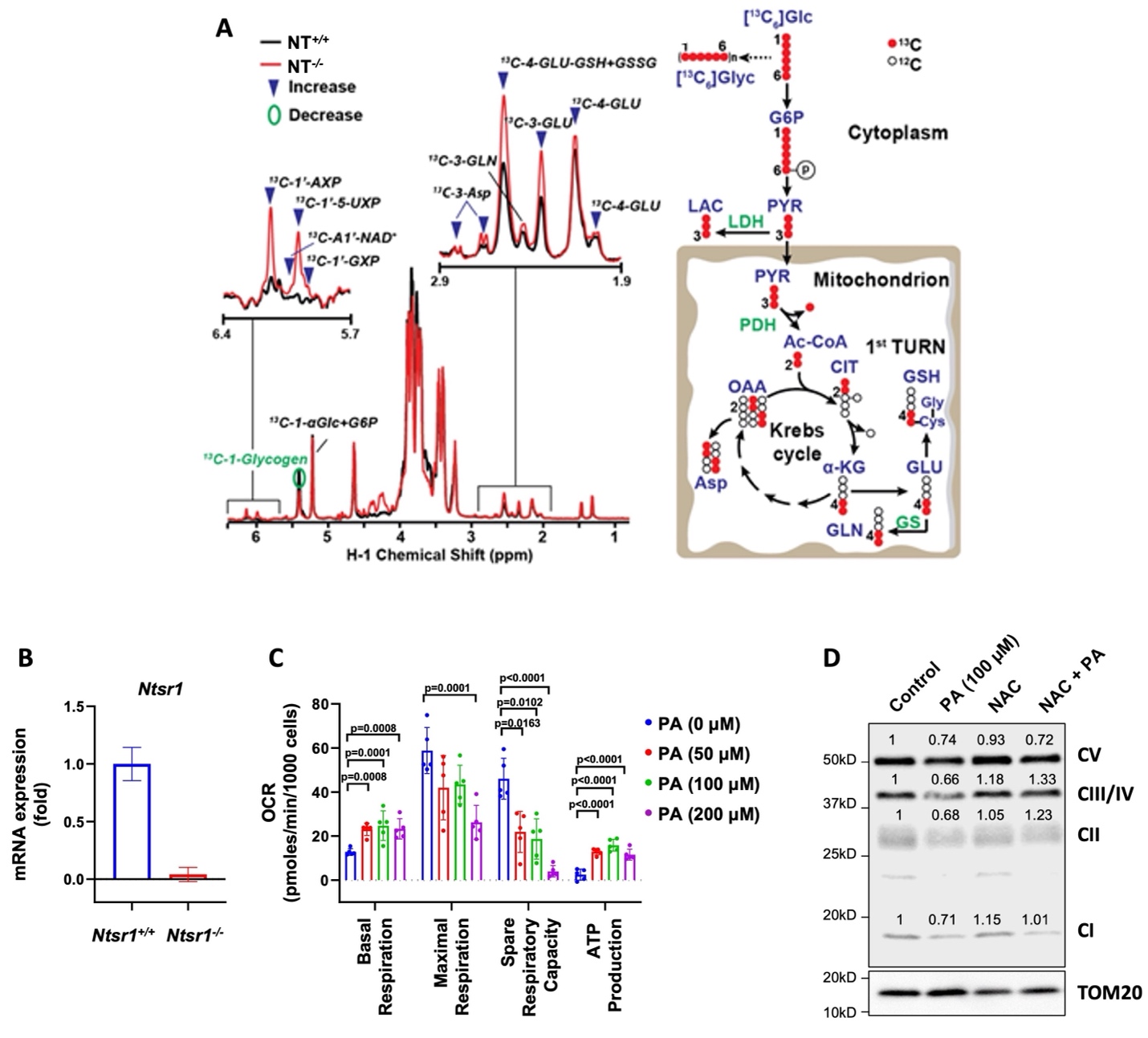
**

**Figure S6. Metabolomics and effect of PA on mitochondrial function. (A)** Results of 1D HSQC NMR analysis using ^13^C_6_-Glucose as a tracer in *Nts^+/+^* and *Nts*^‑/‑^ hepatocytes. N = 1 mouse/genotype; arrows indicate an increase, and the green oval indicates a decrease of respectively labeled metabolites in *Nts*^‑/‑^ livers. Schematic diagram (right) showing the positions of ^13^C after glucose is metabolized through glycolysis and the first turn of Krebs cycle. Numbers refer to carbon positions. **(B)** NTSR1 expression (qPCR) in isolated hepatocytes from *Ntsr1^+/+^* and *Ntsr1*^‑/‑^ mice. Data were normalized to β-actin. **(C)** Primary hepatocytes (C57/BL6) were treated with different concentrations of PA for 16 h, and Mito Stress test was performed to measure mitochondrial function. N = 5 wells/group. Representative data are shown; the experiment was repeated 3 times. **(D)** Effect of N-acetyl cysteine (NAC) treatment on PA-induced decrease in OXPHOS expression in hepatocyte mitochondrial fraction (protein expression was normalized to TOM20 and expressed as fold of Control). Data are expressed as mean ± SD, and p ≤ 0.05 is considered as significant.


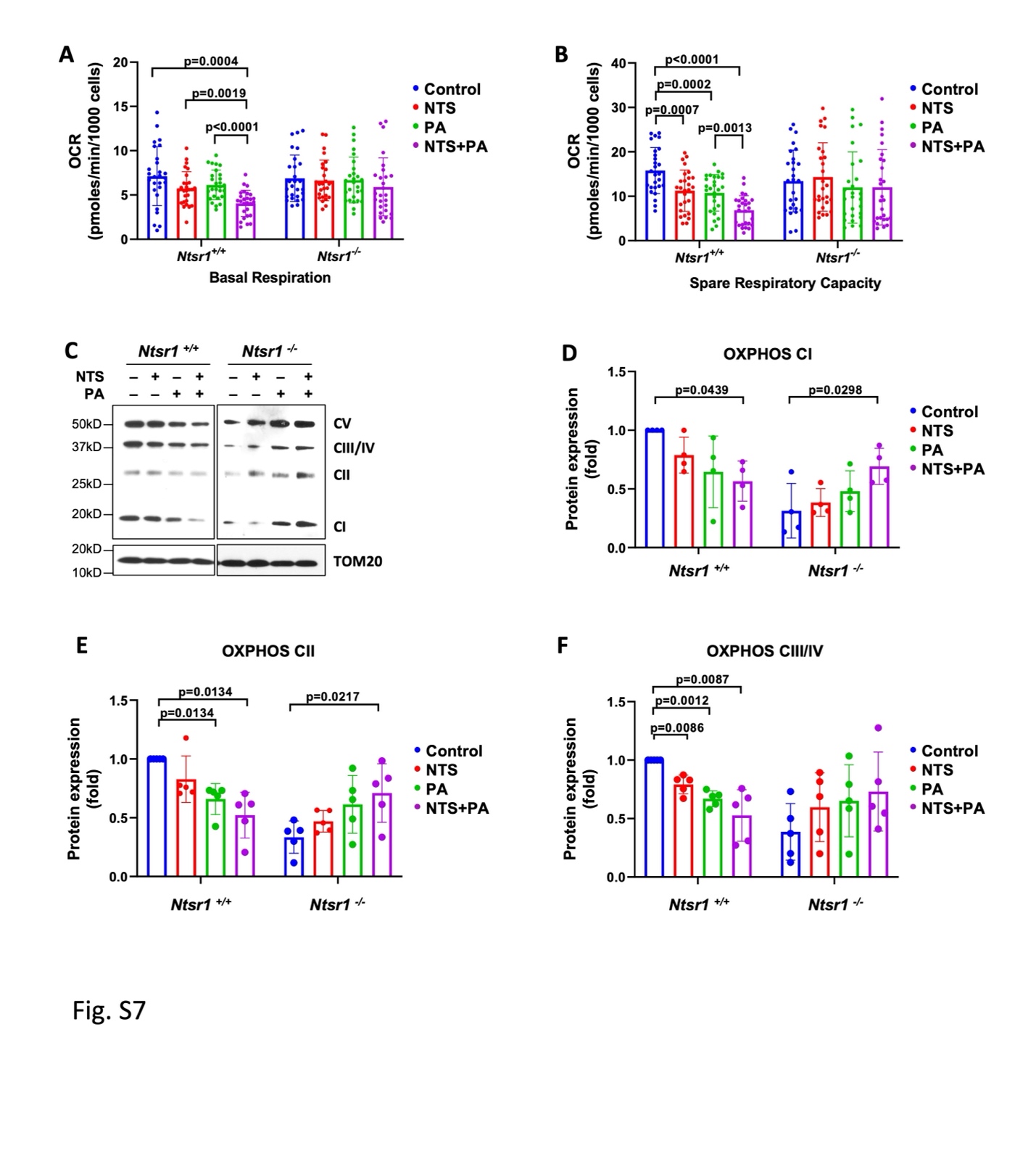


**Figure S7. NTSR1 deficient hepatocytes show better mitochondrial adaptation than NTSR1 wild type hepatocytes**. **(A,B)** Hepatocytes were treated with NTS (10 nM), PA (50 µM) or NTS+PA for 16 h and mitochondrial respiration was measured by Seahorse mito stress test; basal respiration and spare respiratory capacity are shown. N = 27 datapoints from 5 mice/genotype. **(C–F)** OXPHOS complexes I, II and III/IV expression in NTS- and PA-treated hepatocytes were quantified by western blot and normalized to TOM20 expression; N = 4–5 mice/group. TOM20 was used as mitochondrial fraction control. Data are expressed as mean ± SD, and p ≤ 0.05 is considered as significant.


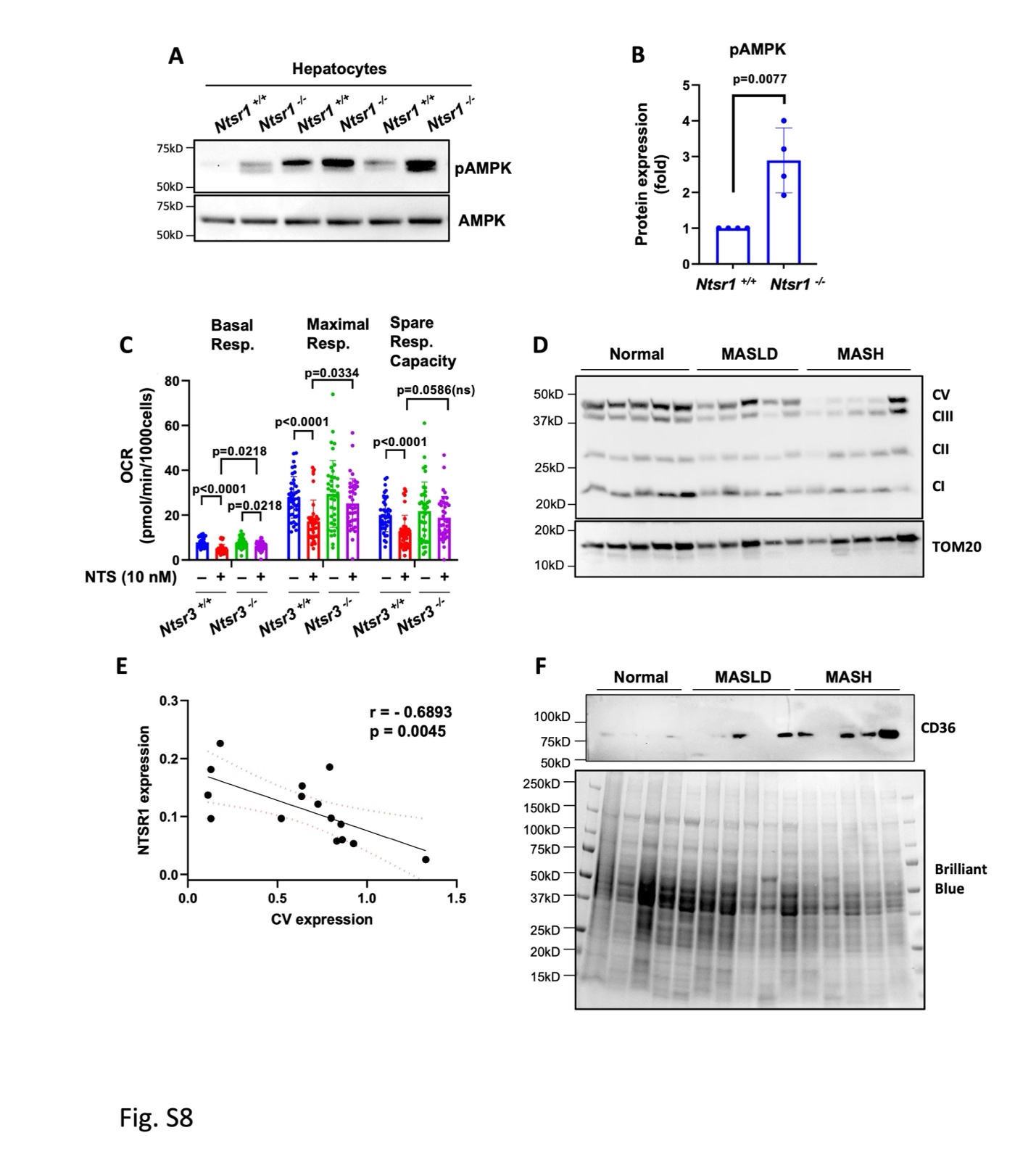


**Figure S8. NTS signaling inhibits AMPK and NTS signaling in human livers. (A)** Representative blot showing AMPK phosphorylation level in hepatocytes isolated from 3 pairs of *Ntsr1^+/+^* and *Ntsr1^-/-^* mice. **(B)** Quantitation of AMPK activation (pAMPK) in hepatocytes. Data were normalized to total AMPK. N=4 pairs of mice. **(C)** Mito stress test showing mitochondrial function in *Ntsr3 ^+/+^* or *Ntsr3 ^-/-^* hepatocytes treated with or without NTS. Data were analyzed from 30 datapoints of 3 mice/genotype. **(D)** Representative western blot showing OXPHOS complexes expression in human livers. TOM20 = loading control; N = 5/group. **(E)** Correlation analyses of NTSR1 and OXPHOS complex V expression in liver samples from human patients. r = Spearman rho; 95% confidence interval lines are shown in red. **(F)** Representative blot showing CD36 expression in human liver samples. Loading control = Brilliant Blue stained gel (same as Fig. 1A); N = 5/group.
