# Supplementary figures and images for: Neurotensin promotes hepatic steatosis by regulating lipid uptake and mitochondrial adaptation in hepatocytes
Source: Cell Death Dis. 2025 Apr 27;16(1):347. doi: 10.1038/s41419-025-07664-3 (PMC12033321; doi:10.1038/s41419-025-07664-3)

# Uncropped Western Blots


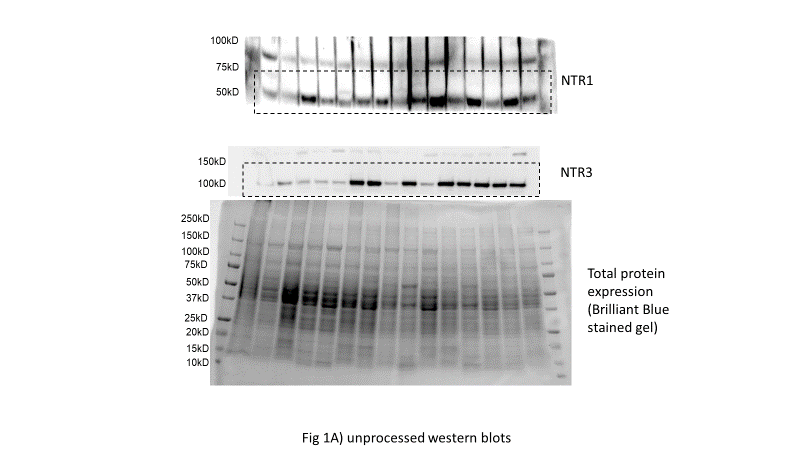


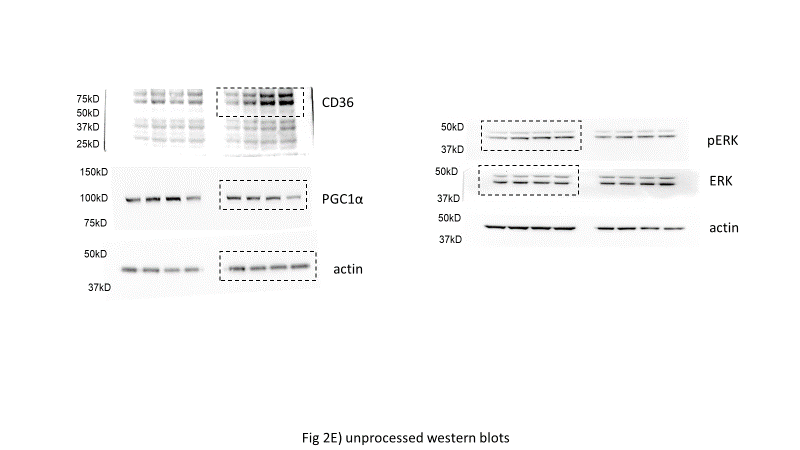


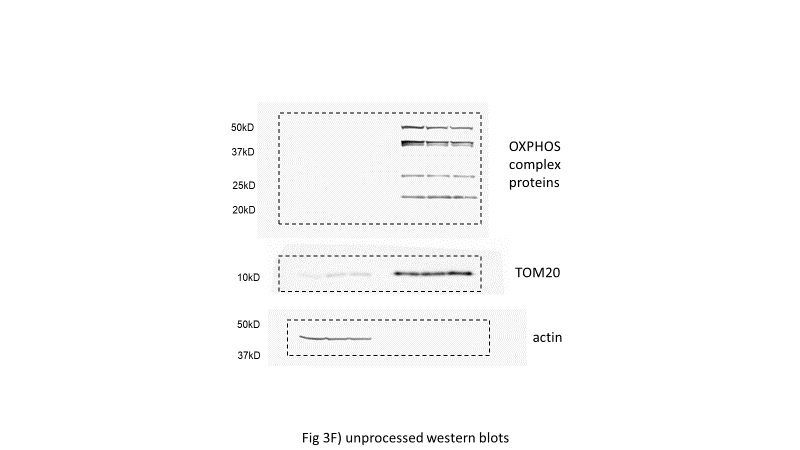


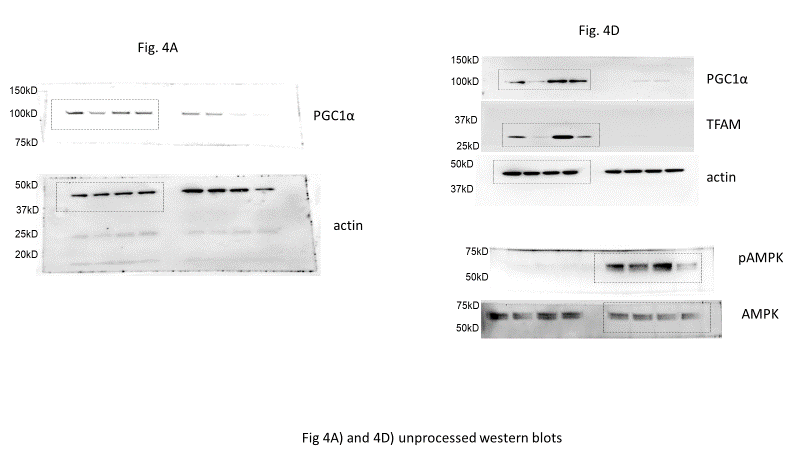


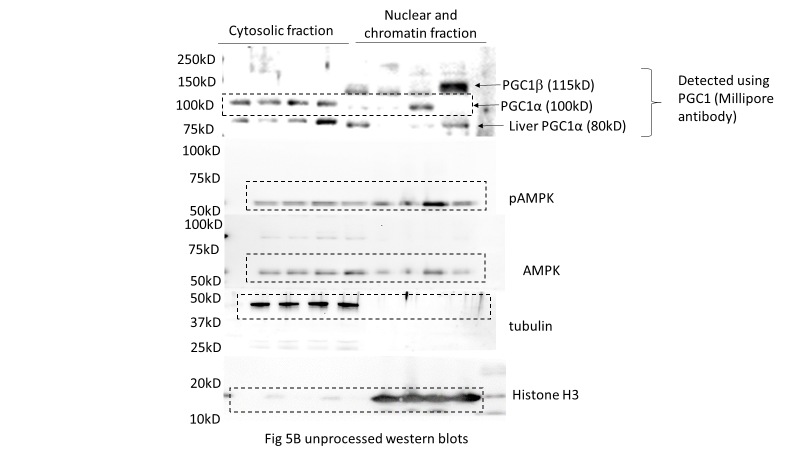


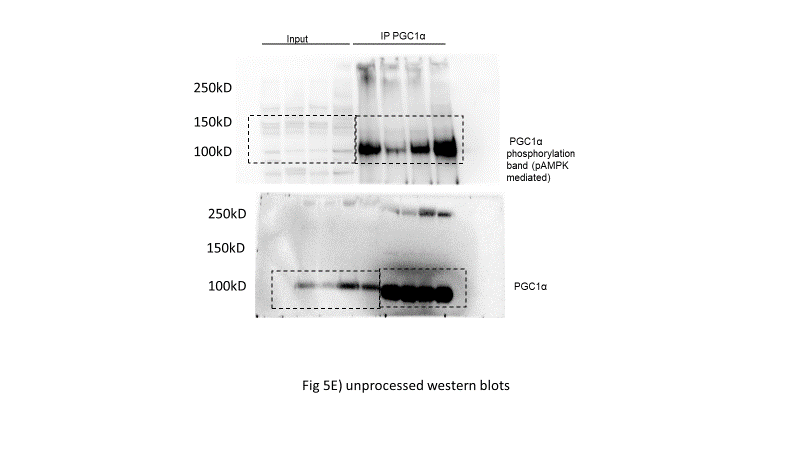


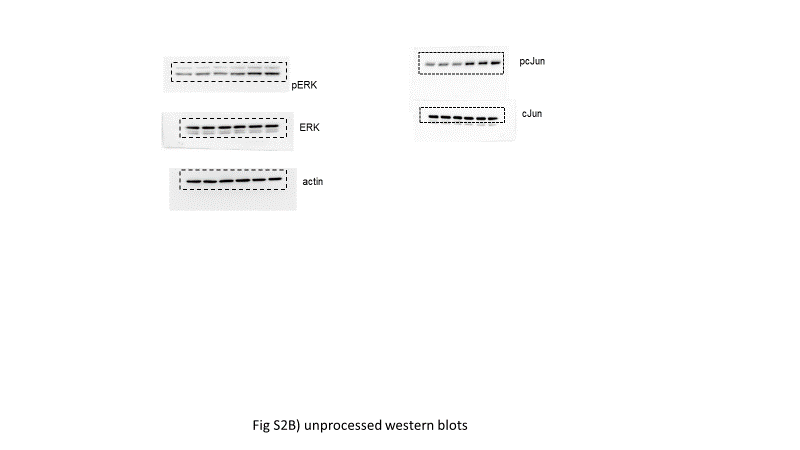


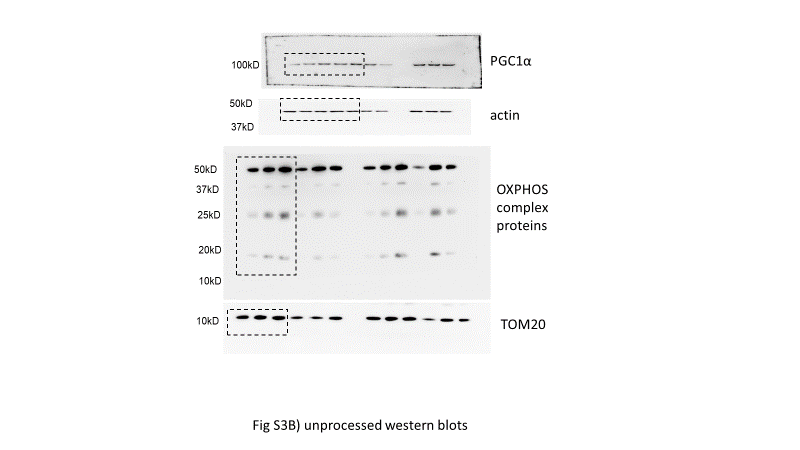


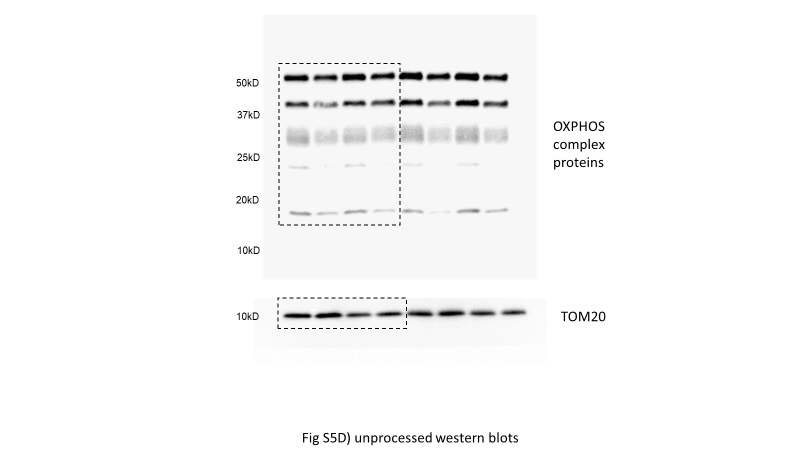


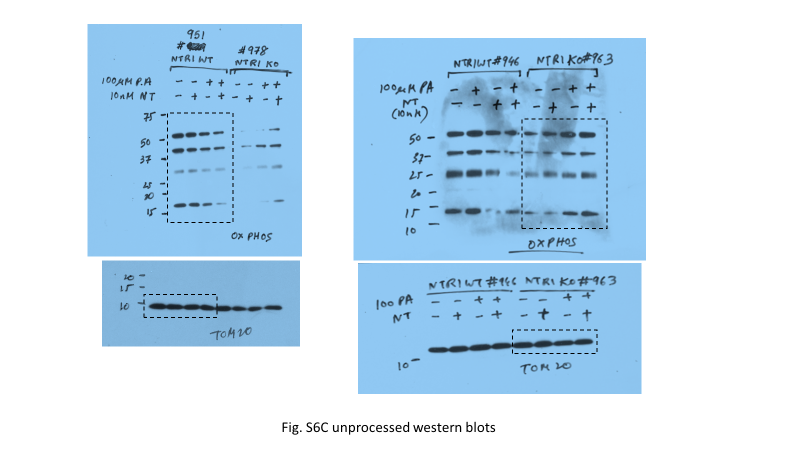


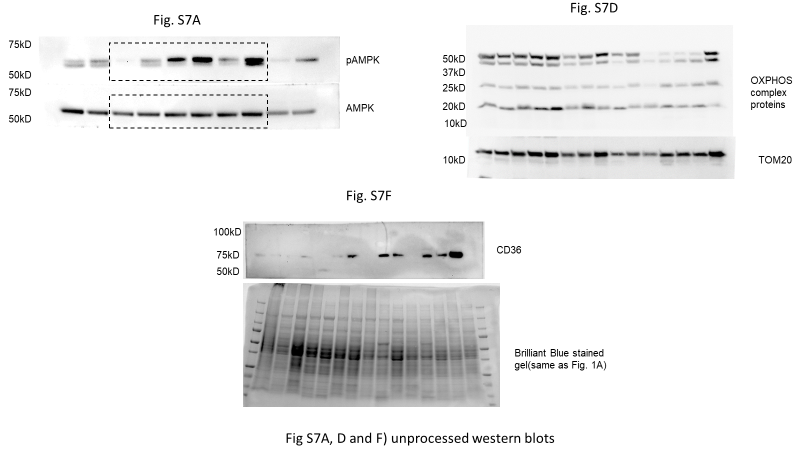

Supplement: Supplementary file 2 — Uncut Western Blots [file 41419_2025_7664_MOESM2_ESM.docx]
